# DNA-Methylation-Based Detection of Urological Cancer in Urine: Overview of Biomarkers and Considerations on Biomarker Design, Source of DNA, and Detection Technologies

**DOI:** 10.3390/ijms20112657

**Published:** 2019-05-30

**Authors:** Louise Katrine Larsen, Guro Elisabeth Lind, Per Guldberg, Christina Dahl

**Affiliations:** 1 Danish Cancer Society Research Center, DK-2100 Copenhagen, Denmark; louisekl@cancer.dk (L.K.L.); perg@cancer.dk (P.G.); 2 Department of Molecular Oncology, Institute for Cancer Research, Oslo University Hospital, the Norwegian Radium Hospital, NO-0424 Oslo, Norway; Guro.Elisabeth.Lind@rr-research.no

**Keywords:** noninvasive detection, DNA methylation biomarkers, bisulfite conversion, urological cancer, bladder cancer, prostate cancer, upper urinary tract cancer, renal cancer

## Abstract

Changes in DNA methylation have been causally linked with cancer and provide promising biomarkers for detection in biological fluids such as blood, urine, and saliva. The field has been fueled by genome-wide characterization of DNA methylation across cancer types as well as new technologies for sensitive detection of aberrantly methylated DNA molecules. For urological cancers, urine is in many situations the preferred “liquid biopsy” source because it contains exfoliated tumor cells and cell-free tumor DNA and can be obtained easily, noninvasively, and repeatedly. Here, we review recent advances made in the development of DNA-methylation-based biomarkers for detection of bladder, prostate, renal, and upper urinary tract cancers, with an emphasis on the performance characteristics of biomarkers in urine. For most biomarkers evaluated in independent studies, there was great variability in sensitivity and specificity. We discuss issues that impact the outcome of DNA-methylation-based detection of urological cancer and account for the great variability in performance, including genomic location of biomarkers, source of DNA, and technical issues related to the detection of rare aberrantly methylated DNA molecules. Finally, we discuss issues that remain to be addressed to fully exploit the potential of DNA-methylation-based biomarkers in the clinic, including the need for prospective trials and careful selection of control groups.

## 1. Introduction

Urological cancers encompass a clinically and molecularly heterogeneous group of neoplasms affecting any region of the urological system. Cancer of the bladder, kidneys, upper urinary tract (ureter and renal pelvis), and prostate are all relatively common and pose specific requirements for diagnosis and follow-up. While kidney and upper urinary tract cancers are detected using imaging techniques, standard work-up for bladder and prostate cancer involves semi-invasive procedures (i.e., cystoscopy and digital rectal examination (DRE), respectively). Cystoscopy is extensively used in clinical practice and poses a significant burden on the healthcare system because it is used as a first-line rule-out test for cancer in patients with relevant symptoms, primarily, hematuria. Other downsides include patient discomfort and anxiety, risk of infectious complications, and high rates of false-positive and false-negative results. Subsequent histopathological assessment of biopsy tissue and surgical resection specimens is the gold standard for cancer diagnosis but also has its limitations, including the subjective evaluation by a pathologist, the need for tissue that is of a certain quality and representative of the tumor, and constraints on sampling frequency. Given these challenges, there is a major unmet need to develop noninvasive methods that could provide clinicians with rapid, objective, and accurate routines for detection of urological cancers.

An important development in cancer care is “liquid biopsy”, which involves the analysis of genetic material or tumor cells shed from primary or metastatic tumors into bodily fluids. A rapidly increasing number of studies have demonstrated the potential of liquid biopsies for a wide range of clinical applications, such as initial diagnosis, early detection, and disease monitoring after therapy [1]. Most of this work involves the analysis of cell-free DNA (cfDNA) circulating in the blood; however, for urological cancers, a more convenient liquid biopsy source is voided urine, which is easily and repeatably accessible and contains exfoliated cells and cfDNA from different sites of the urinary system [2]. The advent of high-sensitivity PCR-based technologies has enabled reliable detection of cancer-specific alterations in urine DNA. Because tumor-derived DNA in urine is often present in a large background of DNA derived from normal cells, the most useful DNA biomarkers are those that provide high specificity for malignancy or premalignancy, including mutations, translocations, gene fusions, and aberrant hypermethylation of specific CpG sites. Among these, DNA hypermethylation events represent the most versatile biomarker type because they are common in most cancers and can be easily assessed using well-established techniques. Furthermore, DNA methylation changes are considered early events in tumorigenesis and thus provide potential biomarkers for early diagnosis [3].

Urine-based DNA tests for urological cancer can be divided into two categories depending on the a priori availability of information on the patient’s tumor DNA. For detection of recurrence and evaluation of treatment response, DNA from the original tumor can be analyzed to identify specific alterations that may serve as “personalized” biomarkers. For other applications, such as initial examination of patients with symptoms of urological cancer, the genetic and epigenetic makeup of the possible tumor is unknown. In these situations, there is a need for a “universal” or “generic” test that can detect, in principle, any cancer. Because no genetic or epigenetic alteration is present in all cases of a urological cancer type, it is necessary to use a combination of biomarkers. The initial assembly of a biomarker panel is facilitated by information about the performance characteristics of individual biomarkers in terms of sensitivity (the true positive rate), specificity (the true negative rate), and predictive values [4]. If the test is used in individuals with unknown disease status to reduce the use of invasive procedures, the most important performance characteristics are sensitivity and negative predictive value (NPV) to achieve the lowest possible rate of false-negative results. As discussed below, specificity is a less well-defined characteristic that varies based on a number of factors, including control populations and the definition of a false-positive result.

Although the promises of DNA-methylation-based detection of cancer have been recognized since the early 2000s [5], including the potential for detection and management of urological cancers [6], only a few DNA methylation biomarkers have been implemented into routine clinical practice. Navigating towards clinical utility is challenging, requiring optimal study designs (representative and large patient series) as well as robustly designed biomarker assays. Here, we provide an overview of current DNA-methylation-based biomarkers for urological cancer, with an emphasis on their performance characteristics in urine. We discuss potential causes of performance variability across studies and other challenges that must be overcome before clinically useful tests can be developed and implemented. Some of these issues have been discussed in detail elsewhere [7,8,9] and are only reviewed briefly here.

## 2. Performance Characteristics of DNA Methylation Biomarkers

To provide an overview of current DNA-methylation-based biomarkers for urine-based detection of urological cancer, we undertook systematic literature searches in PubMed and Embase until February 2019. Details on search strategy, criteria for selection of relevant studies, and data extraction are provided in Supplementary Methods.

### 2.1. Bladder Cancer 

**Figure 1:**
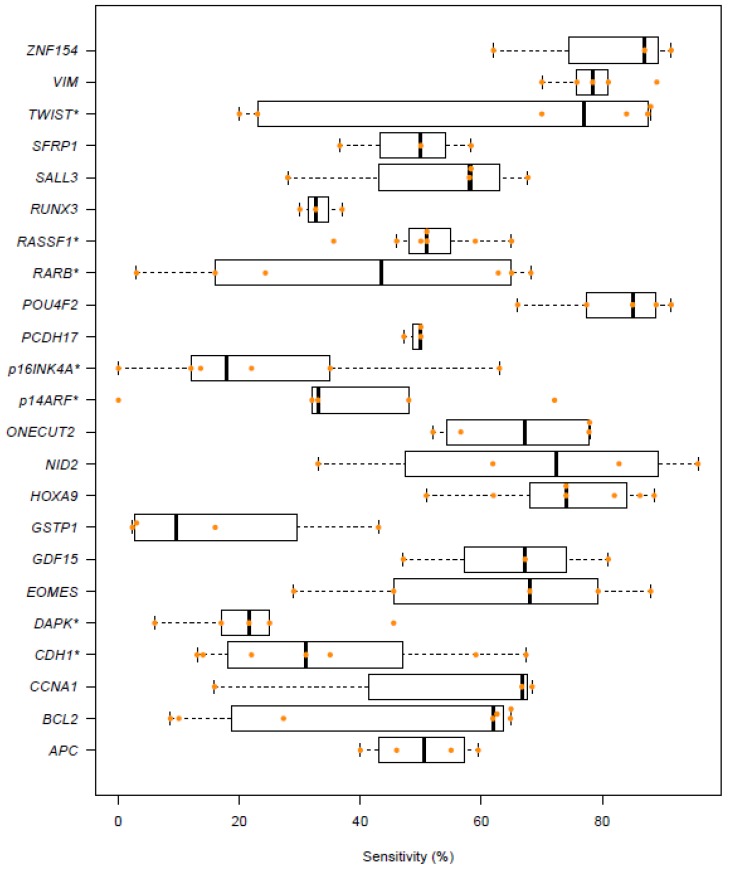
Reported sensitivities of DNA methylation biomarkers for detection of primary bladder cancer. *, Inconsistent nomenclature among studies.

A total of 57 studies met the inclusion criteria (Supplementary Table S1). Fifty-two studies analyzed urine from bladder cancer patients at first diagnosis, using a total of 114 different DNA methylation biomarkers. The sensitivities for 23 biomarkers investigated in more than three studies are shown in Figure 1. Two of these biomarkers each reached a median sensitivity of >80% (*ZNF154* and *POU4F2*) with, however, large variability across studies. The specificities for these biomarkers are shown in Supplementary Figure S1, also demonstrating large interstudy variability. Fifty-two different biomarker combinations, comprising between 2 and 12 individual biomarkers, have been tested for initial diagnosis of bladder cancer. Twenty of these combinations achieved a sensitivity of ≥90% (listed in Table 1).

Eleven studies investigated recurrent bladder cancer, using 18 individual biomarkers and 8 biomarker panels (Table 2). *ZNF154* and *EOMES* achieved the highest sensitivity (94%), however with a specificity of <70%. The biomarker combination with the highest sensitivity (*CFTR, SALL3*, and *TWIST1*; 90%) had a very low specificity (31%). Only one individual biomarker (*TWIST1*) and two panels have been evaluated in more than one study.

### 2.2. Prostate Cancer

**Figure 2:**
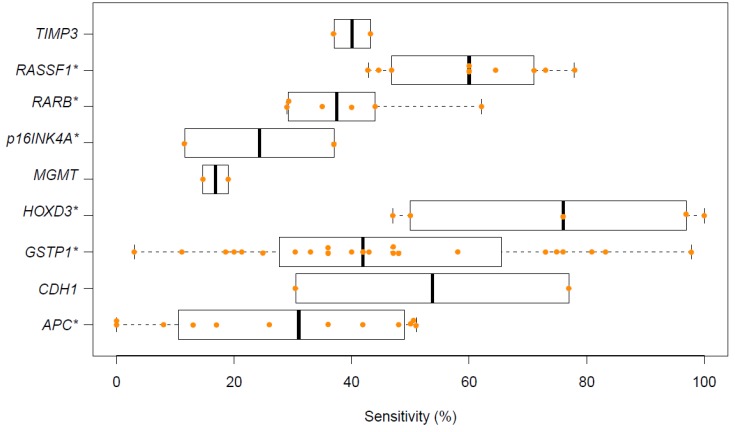
Reported sensitivities of DNA methylation biomarkers for detection of prostate cancer. *, Inconsistent nomenclature among studies.

Twenty-seven studies met the inclusion criteria (Supplementary Table S2). Of these, 26 studies contained data on single biomarker performance (48 different biomarkers), with 9 biomarkers tested in 2 or more studies (shown in Figure 2). *GSTP1* was the most extensively studied biomarker (19 studies). The highest median sensitivity was reported for *HOXD3* (76%); most other biomarkers had sensitivities of <50%. The corresponding specificities for these biomarkers are shown in Supplementary Figure S2. Fifteen studies combined two or more biomarkers, with five studies achieving a sensitivity of ≥90% (Table 3).

### 2.3. Renal Cancer

Five studies met the inclusion criteria (Table 4). Data on single biomarkers were available from four studies for 15 different biomarkers, with sensitivities between 5% and 79% and a generally high specificity (89–100%). *TCF21* was the only biomarker tested in more than one study. Both studies achieved 100% specificity, but the sensitivity varied from 28% to 79%. Three studies evaluated biomarker combinations, with the best performing combination (*VHL, p16, ARF, APC, RASSF1A*, and *TIMP3*) achieving a sensitivity of 88% and a specificity of 100%. A similar sensitivity was reported by combining nine biomarkers (*APC, ARF, CDH1, GSTP1, MGMT, p16, RARB2, RASSF1A*, and *TIMP3*), with no indication of specificity. 

### 2.4. Upper Urinary Tract Cancer

Only two studies met the inclusion criteria, investigating a total of 10 biomarkers (Table 5). *VIM* was the only biomarker investigated in both studies, achieving a sensitivity of 82% and 73% and a specificity of 100% and 61%. *HSPA2* achieved a sensitivity of 83% but with a specificity of only 36%. Among seven biomarker combinations tested, a combination of *VIM* and *GDF15* reached a sensitivity of 91% and a specificity of 100% in a study with 22 cases and 20 healthy controls, whereas in a study with 98 cases and 113 controls with benign urologic conditions, these two biomarkers in combination with *CDH1*, *RASSF1A*, and *HSPA2* only reached a sensitivity of 82% and a specificity of 65%. 

## 3. Factors Affecting Biomarker Performance

The overall conclusion from our review of DNA-methylation-based biomarkers for detection of urological cancer is that there is great variability in sensitivity and specificity across studies. Below, we discuss clinical and technical factors, which can explain this variability and should be considered when designing studies that eventually should lead to the implementation of urine-based tests in the clinic.

### 3.1. Urine Collection and Processing

Urine is a complex biological fluid that contains inorganic salts, organic compounds, and multiple cell types, including leukocytes, urothelial cells, renal cells, and prostate cells. Tumor-derived DNA can be present in both the cellular and cell-free fractions of urine, and the procedures used for collection and processing of DNA will greatly impact the outcome of biomarker analysis. Several sources of DNA have been utilized, including (i) whole urine (containing cellular DNA and cfDNA), (ii) urine sediment obtained by centrifugation (containing cellular DNA), (iii) urine supernatant (containing cfDNA), and (iv) cells obtained by immune capture [44] or filtration [14,45,46]. Because cells and DNA in urine are susceptible to degradation upon storage depending on time and temperature, correct storage is important when urine samples are not processed immediately. One study found that DNA in urine stored at room temperature was stable only upon addition of preserving agents but also found that DNA remained stable without the addition of preservatives for up to 28 days when stored at −20 °C or −80 °C [47]. A confounding factor in many studies is that samples containing DNA of insufficient quantity or quality were excluded from analysis. Such sample selection bias may lead to an overestimation of performance characteristics. 

The vast majority of studies included in this review utilized sedimented urine as the source of DNA. The procedure for collection of urine sediments is simple and inexpensive but has several limitations in addition to storage challenges and processing time, including the co-sedimentation of normal cells and the presence of crystals and substances that may inhibit downstream PCR analyses [48]. A recent study showed that the sensitivity for detection of bladder cancer using *TERT* promoter mutations as a biomarker was higher in sedimented samples compared with cfDNA [2]. However, in leukocyte-rich urine, the sensitivity was higher in cfDNA using next-generation sequencing (NGS), probably because of a higher ratio of tumor-to-wildtype DNA compared with urine sediments. An alternative approach to enriching for tumor DNA is size-based cell selection, utilizing a filter with a pore size that enables capture of tumor cells with the passage of smaller-sized normal cells, at the same time removing inhibitory substances. One study comparing sedimentation and filtration of urine samples from patients with bladder cancer showed a higher sensitivity for filtration, particularly for low-grade tumors [46]. 

Another factor that should be considered when designing urine-based assays for urological cancer is that the concentrations of cells and DNA in urine are not constant. Shedding of cells and release of DNA through apoptosis or necrosis are stochastic and depend on several factors, including the size and location of the tumor. In prostate cancer, higher sensitivities have been achieved after physical manipulation of the prostate, such as massage and DRE. Another approach to increase the sensitivity is repeated urine sampling. In a study of men with high-grade prostate cancer, analysis of urine cells collected by filtration on different days without prior DRE showed a great interday variation in the presence of DNA methylation biomarkers, with some samples giving a false-negative result [49]. A study of patients with small low-grade bladder tumors showed that analysis of pooled urine samples collected over 24 h resulted in a sensitivity of 100%, whereas it was only 75% when a single urine sample was analyzed [50]. 

### 3.2. Bisulfite Treatment, Detection Technologies, and Sample Scoring

With few exceptions, the studies included in this review used treatment of DNA with sodium bisulfite to selectively convert unmethylated cytosines to uracil (leaving methylated cytosines as cytosine). These methylation-dependent C-to-U changes can subsequently be analyzed using PCR-based technologies to ascertain the methylation status [50]. Although the basic protocol for bisulfite conversion is simple and well described, it has a number of limitations that can introduce biases. Treatment of DNA with bisulfite introduces DNA strand breaks and results in highly fragmented single-stranded DNA, leading to degradation of up to 90% of the input DNA [51] and severely reducing the number of molecules effectively available for PCR amplification. The loss of DNA may be further aggravated by incomplete DNA recovery after bisulfite conversion. Recovery depends on the length of input DNA, with higher recovery for high-molecular-weight DNA. In urine, cfDNA has a large component of mononucleosomal (178 bp) DNA, posing specific requirements for extraction procedures [52]. Another limitation inherent to bisulfite treatment is incomplete conversion, which may lead to false-positive results because unconverted unmethylated CpG sites are falsely interpreted as methylated. A comparison of 12 commercially available bisulfite conversion kits showed a large variability in recovery and conversion efficiency [53]. Furthermore, several factors have been shown to affect the technical variability of PCR-based analysis of bisulfite-treated DNA, including the amount of bisulfite-converted template in the PCR, the amount of DNA input in the bisulfite conversion, and storage (bisulfite-converted DNA is less stable than genomic DNA) [54].

A wide range of PCR methods have been used for downstream biomarker evaluation. The most frequently used methods are methylation-specific PCR (MSP) and quantitative MSP (qMSP). Conventional MSP [55] was the method of choice in earlier studies but has also been used in more recent studies. This method is easy to perform and requires no specialized equipment but has several limitations, including the qualitative readout. The most frequently used method in more recent studies is qMSP based on the MethyLight technology [56], which provides a semiquantitative readout. Other methods include pyrosequencing [55], methylation-sensitive single nucleotide primer extension (MS-SnuPE) [57], methylation-sensitive high-resolution melting (MS-HRM) [58], and methylation-specific multiplex ligation-dependent probe amplification (MS-MLPA). [59] A systematic evaluation and comparison of assays for measuring DNA methylation at specific CpG sites was recently conducted by the BLUEPRINT Consortium [60]. Most methods performed well in distinguishing methylated from unmethylated DNA but all had limitations in detecting low-abundant molecules. It is likely that the field will be markedly advanced with the introduction of newer technologies such as NGS and digital PCR, which enable DNA quantification with superior sensitivity and accuracy.

A general limitation in most studies reviewed here was the lack of information about assay performance in terms of limit of blank (LoB), limit of detection (LoD), and limit of quantitation (LoQ), which are critical parameters describing the smallest concentration of a biomarker that can be reliably measured [61]. In most cases, there were no predefined thresholds for interpreting assay signals, and several studies did not indicate the number of positive biomarkers required for scoring a sample positive. 

### 3.3. Genomic Location of Biomarker Assays

The most commonly used strategy to identify and develop new DNA methylation biomarkers is targeting functionally relevant locations, such as CpG islands where methylation affects gene expression. The three biomarkers *TWIST1*, *OTX1*, and *ONECUT2* included in the commercial AssureMDx test, evaluating the risk for bladder cancer in patients with hematuria, are examples of this [11]. Whereas the biomarker for *TWIST1* is located in the gene promoter and associated with loss of gene expression, the assays for *OTX1* and *ONECUT2* are located in regions associated with increased gene expression [8].

Detailed promoter methylation studies have demonstrated that some CpG sites may influence gene expression more than others. This was first shown in 2002, when Deng et al. reported that methylation of CpG sites in a proximal region of *MHL1* was associated with lack of expression, whereas CpG sites in the distal part of the promoter tended to be methylated independently of MLH1 expression [62]. Designing biomarker assays close to the transcription start site generally increases the likelihood of hitting a location where the DNA methylation status will have a functional effect. 

Independent of whether DNA methylation is functionally important or not, detailed knowledge of the methylation pattern of the individual CpG sites (e.g., through TCGA data) in a genomic region of interest is useful prior to biomarker assay design. Methylation density may vary considerably within a genomic region, potentially affecting the sensitivity and specificity of a biomarker assay. From a biomarker perspective, CpG sites that most robustly separate cases from controls and reach the highest sensitivity and specificity (independent of functional effect) would be highly attractive. 

### 3.4. Sensitivity, Specificity, and Control Populations

Most DNA methylation biomarkers reviewed here were originally discovered by analysis of DNA from tumor biopsies, using adjacent tumor-free tissue or normal tissue as control. The sensitivity of a biomarker may here be defined as the proportion of tumors positive for this biomarker. However, a biomarker with high sensitivity in tumor tissue may not necessarily provide the same sensitivity in urine because this will depend on the shedding of tumor cells or cfDNA. Only a few studies have compared urine and tumor tissue from the same patients, suggesting that the sensitivity is generally lower in urine. As larger tumors will shed more material than smaller tumors, sensitivity is highly dependent on the cohort composition, with studies having a higher proportion of advanced cancers achieving higher sensitivity. Only a few studies have evaluated the sensitivity of biomarkers in large prospective studies enrolling patients consecutively and in an unbiased manner.

The specificity of a urinary DNA methylation biomarker is the probability of a negative test result in individuals without cancer. Based on the data compiled in this review, the specificity of DNA methylation biomarkers was relatively high in renal carcinoma (>90%) but generally lower in prostate cancer and recurrent bladder cancer. Although these figures may reflect a true difference in the ability of biomarkers to discriminate between cancer and no cancer, it is important to consider that specificity is affected by choice of control group. In the ideal situation, cases and controls should be age and sex matched. Notably, because epigenetic modifications (including DNA methylation) increase with age, the use of a non-age-matched control group could introduce significant bias. Another important factor is the clinical status of the control group. In many studies, including those on renal carcinoma, the control population consisted of healthy individuals. To evaluate the specificity of a test in a more realistic setting, the control population should consist of individuals with symptoms relevant for the specific cancer. Examples of such control groups include individuals with hematuria (in the case of bladder cancer) and increased prostate-specific antigen (PSA) levels (in the case of prostate cancer). One caveat here is that some patients with a positive urine test may have early cancers or precursor lesions that are molecularly detectable but still undetectable using current scanning or endoscopic procedures. 

None of the biomarkers or biomarker panels for bladder cancer detected recurrence with a sensitivity or specificity of more than 90%, despite better performance of the same biomarkers for detection of primary bladder tumors [13]. The lower sensitivity may be explained by the fact that recurrent tumors are usually smaller than primary tumors and therefore are less prone to shed material into urine [13]. The lower specificity may at least in part be ascribed to challenges in the study design. While control groups for evaluating specificity at first diagnosis of bladder cancer are usually individuals with no prior history of bladder cancer, controls for recurrence are groups of patients showing negative follow-up cystoscopy. It is possible that these patients have residual DNA biomarkers in the urine due to incomplete tumor resection or the emergence of an as-yet undetectable recurrent tumor, thereby resulting in a lower specificity. Longitudinal studies where the patient is his/her own control may be more accurate, at least when it comes to the sensitivity.

## 4. Conclusions

Studies over more than a decade have demonstrated the great potential of DNA methylation biomarkers for urine-based detection of urological cancer. However, the bewildering number of biomarkers currently under evaluation and the great variability in biomarker performance across studies hamper successful translation into clinically useful tests. We have highlighted a number of factors, which directly impact the performance of urinary DNA methylation biomarkers, including technical issues related to the design and implementation of biomarker assays. Guidelines for these procedural issues should be clearly defined to ensure reproducibility and eventually facilitate the development of clinically useful urinary tests for urological cancer.

## Figures and Tables

**Table 1:** DNA-methylation biomarker panels for detection of primary bladder cancer.

Biomarker	Sample Processing	Cases (*n*)	Controls (*n*)	Pathology	Control Population	Method	Sens. (%)	Spec. (%)	Ref.	Year
*SOX1, TJP2, MYOD, HOXA9_1, HOXA9_2, VAMP8, CASP8, SPP1, IFNG, CAPG, HLADPA1, RIPK3* (Positive when six or more markers are present)	Sedimentation	73	18	Ta-T4, Grade 1–3	Healthy	Pyrosequencing	100	100	[10]	2013
*POU4F2, PCDH17, GDF15*	Sedimentation	72	92	Ta-T4, LG, HG	Mixed urologic diseases and healthy	qMSP	97	75	[11]	2016
*ZNF671, SFRP1, IRF8*	Sedimentation	26	19	Ta-T4, LG, HG	Noncancer, not specified	qMSP	96	84	[12]	2015
*POU4F2, EOMES*	Sedimentation	72	92	Ta-T4, LG, HG	Mixed urologic diseases and healthy	qMSP	96	88	[11]	2016
*TWIST1, NID2*	Sedimentation	24	15	Ta-T3, LG, HG	Mixed urologic diseases, and healthy	MSP	96	93	[13]	2013
*BCL2, EOMES, VIM, SALL3, CCNA1, HOXA9, POU4F2*	Filtration (8 µm)	33	26	Ta-T2, LH, HG, PUNLMP	Mixed urologic diseases, negative findings	qMSP	94		[14]	2015
*TWIST1, NID2*	Sedimentation	35	57	Ta-T2, LG, HG	Mixed urologic diseases	qMSP	94	91	[15]	2010
*VIM, TMEFF2, GDF15*	Sedimentation	51	20	Not Specified	Healthy	qMSP	94	100	[16]	2010
*VIM, TMEFF2, GDF15, HSPA2*	Sedimentation	51	20	Not Specified	Healthy	qMSP	94	100	[16]	2010
*SALL3, CFTR, ABCC6, HPR1, RASSF1A, MT1A, ALX4, CDH13, RPRM, MINT1, BRCA1*	Sedimentation	132	23	Stage 0a-IV	Mixed urologic diseases	MSP	92	87	[17]	2007
*SALL3, CFTR, MT1A, HPP1, ABCC6, RASSF1A, CDH13, RPRM, MINT1, BRCA1, SFRP1*	Sedimentation	82	15	Stage pTa-IV	Mixed urologic diseases	MSP	92	73	[18]	2009
*POU4F2, PCDH17*	Sedimentation	58	90	Ta-T4, LG, HG	Mixed urologic diseases and healthy	qMSP	91	93	[11]	2016
*POU4F2, PCDH17, GDF15*	Sedimentation	58	90	Ta-T4, LG, HG	Mixed urologic diseases and healthy	qMSP	91	88	[11]	2016
*p14ARF, p16INK4A, RASSF1A, DAPK, APC*	Sedimentation	113		≥T1, PUNLMP, Grade 1–3	Healthy	MSP	91		[19]	2017
*SEPTIN9, SLIT2*	Filtration (11 µm)	167	105	Ta-T1 (NMIBC), LG, HG	Patients with negative cystoscopy (hematuria)	qMSP	91	71	[20]	2016
*RARβ, DAPK, CDH1, p16*	Sedimentation	22	17	NMIBC-MIBC, Grade 1–3	Healthy	MSP	91	76	[21]	2002
*HOXA9, PCDH17, POU4F2, ONECUT2*	Sedimentation	Not specified	Not specified	Ta-T4, LG, HG, PUNLMP	Mixed urologic diseases, hematuria	qMSP	91	73	[22]	2018
*HS3ST2, SEPTIN9, SLIT2*	Filtration (11 µm)	167	105	Ta-T1 (NMIBC), LG, HG	Patients with negative cystoscopy (hematuria)	qMSP	90	75	[20]	2016
*HS3ST2, SLIT2*	Filtration (11 µm)	167	105	Ta-T1 (NMIBC), LG, HG	Patients with negative cystoscopy (hematuria)	qMSP	90	34	[20]	2016
*HS3ST2, SEPTIN9*	Filtration (11 µm)	167	105	Ta-T1 (NMIBC), LG, HG	Patients with negative cystoscopy (hematuria)	qMSP	90	72	[20]	2016
*SALL3, CFTR, MT1A, HPP1, ABCC6, RASSF1A, CDH13, RPRM, MINT1, BRCA1*	Sedimentation	82	15	Stage Ta-IV	Mixed urologic diseases	MSP	90	80	[18]	2009
*ONECUT2, VIM, SALL3, CCNA1, BCL2, EOMES*	Filtration (8 µm)	99	376	Ta-T4, LG, HG, PUNLMP	Macroscopic hematuria, no malignancy	qMSP	90	89	[23]	2016

MSP = methylation-specific PCR, qMSP = quantitative MSP, HG = high grade, LG = low grade, PUNLMP = papillary urothelial neoplasm of low malignant potential, NMIBC = nonmuscle invasive bladder cancer, MIBC = muscle invasive bladder cancer.

**Table 2:** DNA-methylation biomarkers and biomarker panels for detection of recurrent bladder cancer.

Biomarker	Sample Processing	Cases (*n*)	Controls (*n*)	Pathology	Control Population	Method	Sens. (%)	Spec. (%)	Ref.	Year
*APC*	Sedimentation	15	25	Ta-T1	No recurrence	qMSP	27	80	[24]	2008
*BCL2*	Sedimentation	15	25	Ta-T1	No recurrence	qMSP	13	96		2008
*DAPK*	Sedimentation	15	25	Ta-T1	No recurrence	qMSP	0	96		2008
*CDH1*	Sedimentation	15	25	Ta-T1	No recurrence	qMSP	7	84		2008
*EDNRB*	Sedimentation	15	25	Ta-T1	No recurrence	qMSP	20	80		2008
*EOMES*	Sedimentation	139	67	Ta-T1, Grade 1–3	Mixed urologic diseases	qMSP	94	55	[25]	2012
*HOXA9*	Sedimentation	139	67	Ta-T1, Grade 1–3	Mixed urologic diseases	qMSP	93	55		2012
*IGFBP*	Sedimentation	15	25	Ta-T1	No recurrence	qMSP	20	84	[24]	2008
*MGMT*	Sedimentation	15	25	Ta-T1	No recurrence	qMSP	20	92		2008
*NID2*		48	275	Ta-T3, Grade 1–3	No recurrence	MSP	46	90	[26]	2012
*POU4F2*	Sedimentation	139	67	Ta-T1, Grade 1–3	Mixed urologic diseases	qMSP	88	64	[25]	2012
*RASSF1A*	Sedimentation	15	25	Ta-T1	No recurrence	qMSP	50	32	[24]	2008
*TERT*	Sedimentation	15	25	Ta-T1	No recurrence	qMSP	13	100		2008
*TNFRSF25*	Sedimentation	15	25	Ta-T1	No recurrence	qMSP	40	56		2008
*TWIST1*	Sedimentation	139	67	Ta-T1, Grade 1–3	Mixed urologic diseases	qMSP	90	43	[25]	2012
*TWIST1*		48	275	Ta-T3, Grade 1–3	No recurrence	MSP	75	69	[26]	2012
*VIM*	Sedimentation	139	67	Ta-T1, Grade 1–3	Mixed urologic diseases	qMSP	90	59	[25]	2012
*WIF1*	Sedimentation	15	25	Ta-T1	No recurrence	qMSP	20	76	[24]	2008
*ZNF154*	Sedimentation	139	67	Ta-T1, Grade 1–3	Mixed urologic diseases	qMSP	94	67	[25]	2012
*APC_a, TERT_a, TERT_b, EDNRB*	Sedimentation	65	29	Ta-T4, Grade 0–3	No recurrence	MS-MLPA	72	55	[27]	2012
*APC_a, TERT_a, TERT_b, EDNRB*	Sedimentation	49	60	Ta-T1, Grade 1–3	No recurrence	MS-MLPA	63	58		2012
*APC_a, TERT_a, TERT_b, EDNRB*	Sedimentation	68	91	Ta-T1, Grade 1–3	No BC	MS-MLPA				2012
*CFTR, SALL3, TWIST1*	Sedimentation	173	285	Ta-T1	Ta-T1	Pyrosequencing	90	31	[28]	2018
*HS3ST2, SLIT2, SEPTIN9*	Filtration (11 µm)	72	86	Ta-T4, LG, HG	Ta-T4, LG, HG	qMSP			[20]	2016
*miR-9-3, miR124-2, miR-124-3, miR-137*	Sedimentation	25	107	Ta-T1	No recurrence	Pyrosequencing	62	74	[29]	2018
*OTX1, ONECUT2, OSR1*	Sedimentation	95	40	NMIBC, Grade 1–3, (recurrence)	No recurrence	SNaPshot	74	Fixed = 90%	[30]	2013
Panel consisting of 41 sequences	Sedimentation	136		≥Ta, LG, HG	Mixed urologic diseases, and healthy	MS-MLPA			[31]	2013
*RASSF1A, ECAD, APC, DAPK, MGMT, BCL2, TERT, EDNRB, WIF1, TNFRSF25. IGFBP*	Sedimentation	15	25	Ta-T1	No recurrence	qMSP	86	8	[24]	2008
*SOX1, IRAK3, L1-MET* (*L1-MET* hypomethylated)	Sedimentation	29	54	Ta-T1, LG, HG	Ta-T1, LG, HG	Pyrosequencing	86	89	[32]	2014
*SOX1, IRAK3, L1-MET* (*L1-MET* hypomethylated)	Sedimentation	134	25	Ta-T1, LG, HG	Ta-T1, LG, HG	Pyrosequencing	80	97		2014

MSP = methylation specific PCR, qMSP = quantitative MSP, HG = high grade, LG = low grade, NMIBC = nonmuscle invasive bladder cancer, BC = bladder cancer, MS-MLPA = methylation-specific multiplex ligation-dependent probe amplification.

**Table 3:** DNA-methylation biomarker panels for detection of prostate cancer.

Biomarkers	Urine Collection	Sample Processing	Cancer (*n*)	Controls (*n*)	Pathology	Control Population	Method	Sens. (%)	Spec. (%)	Ref.	Year
*GSTP1, RARβ2, APC, miR-34b/c + miR-193b*	Morning	Sedimentation	87	32	T2-T3b	Asymptomatic donors	qMSP	100	75	[33]	2018
*TGFB2, HOXD3, APC*	Post DRE	Sedimentation	10	5	Organ confined	Cancer free (not further specified)	qMSP	100	60	[34]	2014
*EDNRB, APC, GSTP1*	Post DRE/biopsy	Sedimentation	12	5	GS 6–7	Biopsy Negative	MSP	100	40	[35]	2006
*miR-34b/c + miR-193b*	Morning	Sedimentation	87	32	T2-T3b	Asymptomatic donors	qMSP	95	84	[33]	2018
*GSTP1, RARβ2, APC*	Morning	Sedimentation	87	32	T2-T3b	Asymptomatic donors	qMSP	94	84		2018
≥6 positive of 19 markers	First Void	Sedimentation	32	35	GS 6–10	Negative biopsy results	Nested qMSP	94	71	[36]	2018
*miR-34b/c + miR-193b*	No DRE	Sedimentation	95	46	GS ≥ 6	No urological malignancy, healthy	qMSP	91	98	[37]	2017

MSP = methylation specific PCR, qMSP = quantitative MSP, DRE = digital rectal examination, GS = Gleason score.

**Table 4:** DNA-methylation biomarkers and biomarker panels for detection of renal cancer.

Biomarker	Sample Processing	Cancer (*n*)	Controls (*n*)	Pathology	Control Population	Method	Sens. (%)	Spec. (%)	Ref.	Year
*APC*	Sedimentation	26	91	Not specified	Various conditions, malignant and nonmalignant	qMSP	38	96	[38]	2004
*ARF*	Sedimentation	26	91	Not specified	Various conditions, malignant and nonmalignant	qMSP	31	100		2004
*CDH1*	Sedimentation	26	91	Not specified	Various conditions, malignant and nonmalignant	qMSP	38	95		2004
*GDF15*	Sedimentation	19	20	Not specified	Healthy	qMSP	5	100	[16]	2010
*GSTP1*	Sedimentation	26	91	Not specified	Various conditions, malignant and nonmalignant	qMSP	15	100	[38]	2004
*HSPA2*	Sedimentation	19	20	Not specified	Healthy	qMSP	11	100	[16]	2010
*MGMT*	Sedimentation	26	91	Not specified	Various conditions, malignant and nonmalignant	qMSP	8	100	[38]	2004
*p16*	Sedimentation	26	91	Not specified	Various conditions, malignant and nonmalignant	qMSP	35	100		2004
*PCDH17*	Sedimentation	50	48	Not specified	Healthy	qMSP	20	100	[39]	2011
*RARB2*	Sedimentation	26	91	Not specified	Various conditions, malignant and nonmalignant	qMSP	31	91	[38]	2004
*RASSF1A*	Sedimentation	26	91	Not specified	Various conditions, malignant and nonmalignant	qMSP	65	89		2004
*TCF21*	Sedimentation	33	15	Grades I–IV	Healthy	Pyrosequencing	79	100	[40]	2016
*TCF21*	Sedimentation	50	48	Not specified	Healthy	qMSP	28	100	[39]	2011
*TIMP3*	Sedimentation	26	91	Not specified	Various conditions, malignant and nonmalignant	qMSP	46	91	[38]	2004
*TMEFF2*	Sedimentation	19	20	Not specified	Healthy	qMSP	11	100	[16]	2010
*VIM*	Sedimentation	19	20	Not specified	Healthy	qMSP	5	100		2010
*PCDH17, TCF21*	Sedimentation	50	48	Not specified	Healthy	qMSP	32	100	[39]	2011
*APC, ARF, CDH1, GSTP1, MGMT, P16, RAR-β2, RASSF1A, TIMP3*	Sedimentation	26	91	Not specified	Various conditions, malignant and nonmalignant	qMSP	88		[38]	2004
*VHL, p16/cdkn2a, p14ARF, APC, RASSF1A, Timp-3*	Sedimentation	50	24	T1–T3	Healthy, renal stones, benign renal cysts	MSP	88	100	[41]	2004

MSP = methylation specific PCR, qMSP = quantitative MSP.

**Table 5:** DNA methylation biomarkers and biomarker panels for detection of upper urinary tract tumors.

Biomarkers	Sample Processing	Cancer (*n*)	Controls (*n*)	Pathology	Control Population	Method	Sens. (%)	Spec. (%)	Ref	Year
*ABCC6*	Sedimentation	98	113	Not specified	Benign urologic conditions	MSP	44	54	[42]	2018
*BRCA1*	Sedimentation	98	113	Not specified	Benign urologic conditions	MSP	26	58		2018
*CDH1*	Sedimentation	98	113	Not specified	Benign urologic conditions	MSP	28	98		2018
*GDF15*	Sedimentation	98	113	Not specified	Benign urologic conditions	MSP	30	90		2018
*HSPA2*	Sedimentation	98	113	Not specified	Benign urologic conditions	MSP	83	36		2018
*RASSF1A*	Sedimentation	98	113	Not specified	Benign urologic conditions	MSP	48	73		2018
*SALL3*	Sedimentation	98	113	Not specified	Benign urologic conditions	MSP	23	80		2018
*THBS1*	Sedimentation	98	113	Not specified	Benign urologic conditions	MSP	74	25		2018
*TMEFF2*	Sedimentation	98	113	Not specified	Benign urologic conditions	MSP	67	40		2018
*VIM*	Sedimentation	98	113	Not specified	Benign urologic conditions	MSP	73	61		2018
*VIM*	Sedimentation	22	20	Not specified	Healthy	qMSP	82	100	[43]	2014
*CDH1, VIM*	Sedimentation	98	113	Not specified	Benign urologic conditions	MSP	82	60	[42]	2018
*CDH1, VIM, RASSF1A*	Sedimentation	98	113	Not specified	Benign urologic conditions	MSP	82	60		2018
*CDH1, VIM, RASSF1A, HSPA2*	Sedimentation	98	113	Not specified	Benign urologic conditions	MSP	85	59		2018
*CDH1, VIM, RASSF1A, HSPA2, GDF15*	Sedimentation	98	113	Not specified	Benign urologic conditions	MSP	82	65		2018
*CDH1, VIM, RASSF1A, HSPA2, GDF15, TMEFF2*	Sedimentation	98	113	Not specified	Benign urologic conditions	MSP	82	68		2018
*VIM, GDF15*	Sedimentation	22	20	Papillary, invasive, LG, HG	Healthy, renal cell carcinoma, prostate carcinoma	qMSP	91	100	[43]	2014
*VIM, GDF15, TMEFF2*	Sedimentation	22	20	Papillary, invasive, LG, HG	Healthy, renal cell carcinoma, prostate carcinoma	qMSP	91	100		2014

MSP = methylation specific PCR, qMSP = quantitative MSP, HG = high grade, LG = low grade.

## Supplementary Materials

Supplementary materials can be found at https://www.mdpi.com/1422-0067/20/11/2657/s1. References [63,64,65,66,67,68,69,70,71,72,73,74,75,76,77,78,79,80,81,82,83,84,85,86,87,88,89,90,91,92,93,94,95,96,97,98,99,100,101,102,103,104,105,106,107,108,109,110,111] are cited in the supplementary materials.
