# Supplementary material for: DNA-Methylation-Based Detection of Urological Cancer in Urine: Overview of Biomarkers and Considerations on Biomarker Design, Source of DNA, and Detection Technologies
Source: Int J Mol Sci. 2019 May 30;20(11):2657. doi: 10.3390/ijms20112657 (PMC6600406; doi:10.3390/ijms20112657)
Supplement: Supplementary file 1 [file ijms-20-02657-s001.pdf]

Supplementary Methods

Literature Search

A systematic search was conducted in the PubMed and Embase databases up to February 2019. Search terms used were: "(*((Bladder cancer) OR urothelial carcinomas) AND methylation) AND urine*", yielding 169 and 239 articles from PubMed and Embase, respectively; "(*(prostate cancer) AND methylation) AND urine*", yielding 91 and 162 articles from PubMed and Embase, respectively; "(*((Upper urinary tract carcinoma) OR Upper urinary tract cancer) AND methylation) AND urine*", yielding 9 and 0 articles from PubMed and Embase, respectively; and "(*((Kidney cancer) OR Renal cancer) AND methylation) AND urine*", yielding 40 and 22 articles from PubMed and Embase, respectively.

Selection of Studies

One author (LKL) screened all published original articles appearing in the above searches for eligibility. Studies using DNA methylation biomarkers for detection of bladder cancer, prostate cancer, kidney cancer, or upper urinary tract cancer in urine were considered eligible. Studies were excluded if they were not original research papers; used a language other than English; or did not report biomarker performance in terms of sensitivity, specificity, or area under the curve (AUC); or reported the performance of DNA methylation markers only in combination with other factors (such as other DNA mutation markers and clinical data). Meeting the inclusion criteria were 57, 27, 2, and 5 articles for bladder cancer, prostate cancer, upper urinary tract cancer, and kidney cancer, respectively.

Data Extraction

Two authors (LKL and CD) independently extracted data from the selected studies and disagreements were discussed until agreement was reached. The data extracted from the articles were obtained both from the primary article and supplementary data and included gene name, reference, study year, number of cases and controls, testing or validation, source of urine, urine processing method, first diagnosis or recurrence, analysis technique, sensitivity, and specificity.

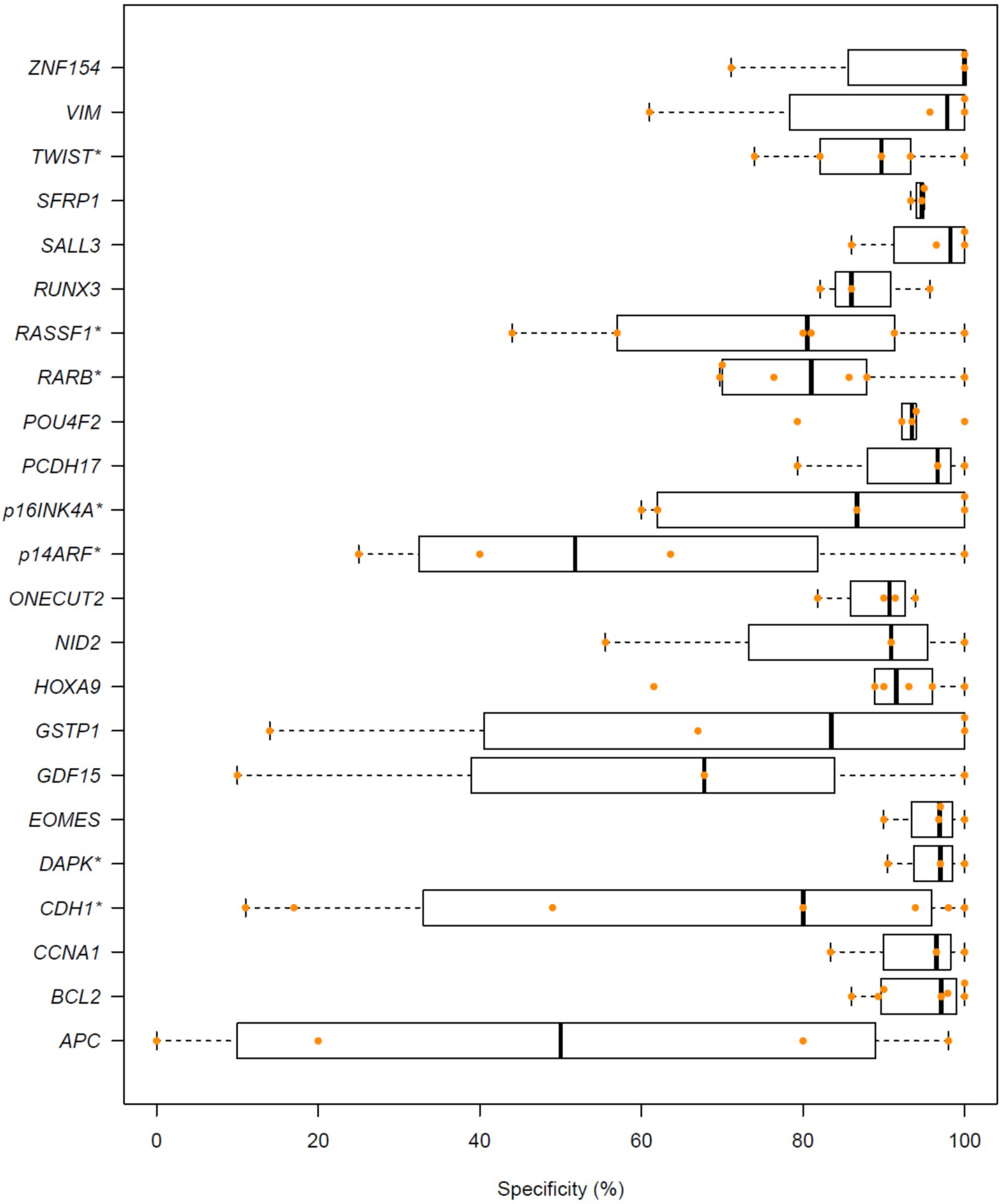

Supplementary Figure S1. Reported specificities of DNA-methylation biomarkers for detection of primary bladder cancer. *, Inconsistent nomenclature among studies.

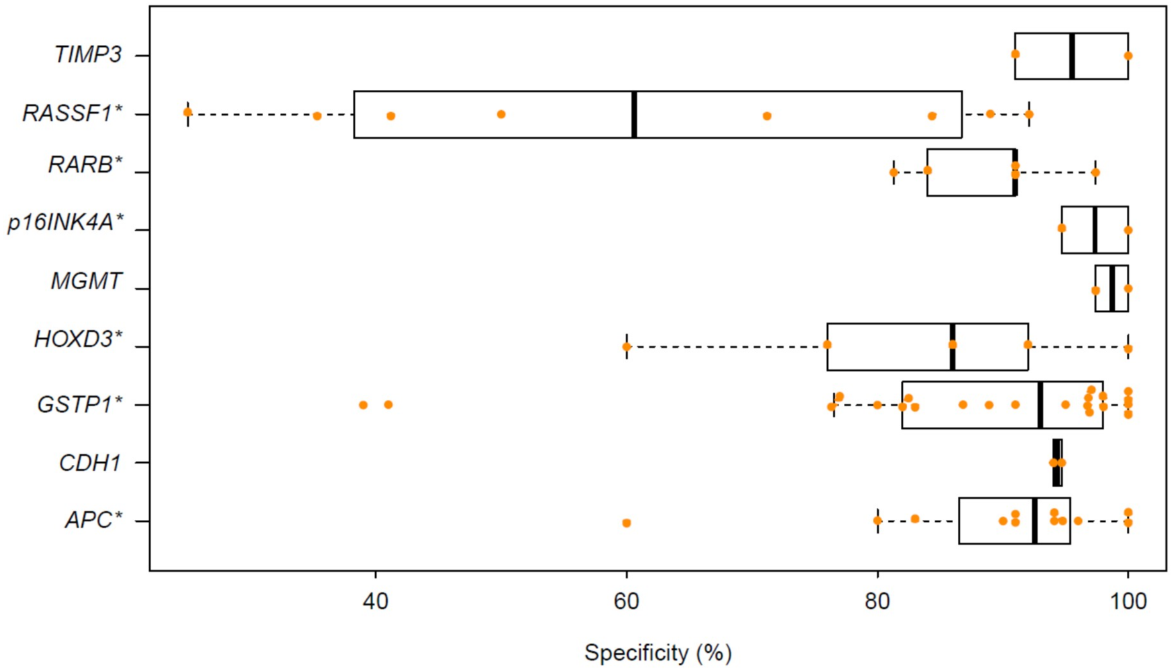

Supplementary Figure S2. Reported specificities of DNA-methylation biomarkers for detection of prostate cancer. *, Inconsistent nomenclature among studies.

Supplementary Table S1. Bladder cancer.

Biomarker	Primary/Recurrence	Dataset	Sample Processing	Cases (n)	Controls (n)	Pathology	Control Population	Method	Sens. (%)									Spec. (%)	AUC	Ref.	Year
									Ta	Cis/tis	T1	≥T2	Ta-T1	LG/G2	HG/G3	PUNLMP/G1	Overall	Overall			
TWIST1, NID2	Primary	Training	Sedimentation	48	121	Ta-T2, LG, HG	Mixed Urologic Disease	q-MSP	80	100	100	83		80	91		88	94		[15]	2010
TWIST1, NID2	Primary	Validation	Sedimentation	35	57	Ta-T2, LG, HG	Mixed Urologic Disease	q-MSP	88	100	100	100		89	100		94	91			2010
SOX1, IRAK3, L1-MET (L1-MET hypomethylated)	Recurrent	Training	Sedimentation	29	54	Ta-T1, LG, HG	Ta-T1, LG, HG	Pyrosequencing									86	89	0.90	[32]	2014
SOX1, IRAK3, L1-MET (L1-MET hypomethylated)	Recurrent	Validation	Sedimentation	134	25	Ta-T1, LG, HG	Ta-T1, LG, HG	Pyrosequencing									80	97	0.95		2014
EOMES	Primary	Training	Sedimentation	58	90	Ta-T4, LG, HG	Mixed urologic diseases and healthy	qMSP									79.31	90.00	0.906	[11]	2016
GDF15	Primary	Training	Sedimentation	58	90	Ta-T4, LG, HG	Mixed urologic diseases and healthy	qMSP									67.24	67.78	0.711		2016
NID2	Primary	Training	Sedimentation	58	90	Ta-T4, LG, HG	Mixed urologic diseases and healthy	qMSP									82.76	55.56	0.703		2016
PCDH17	Primary	Training	Sedimentation	58	90	Ta-T4, LG, HG	Mixed urologic diseases and healthy	qMSP									50.00	96.67	0.813		2016
POU4F2	Primary	Training	Sedimentation	58	90	Ta-T4, LG, HG	Mixed urologic diseases and healthy	qMSP									91.38	92.22	0.921		2016
TCF21	Primary	Training	Sedimentation	58	90	Ta-T4, LG, HG	Mixed urologic diseases and healthy	qMSP									86.21	82.22	0.910		2016
ZNF154	Primary	Training	Sedimentation	58	90	Ta-T4, LG, HG	Mixed urologic diseases and healthy	qMSP									91.38	71.11	0.892		2016

POU4F2, EOMES	Primary	Training	Sedimentation	58	90	Ta-T4, LG, HG	Mixed urologic diseases and healthy	qMSP								87.93	91.11	0.930		2016
POU4F2, PCDH17	Primary	Training	Sedimentation	58	90	Ta-T4, LG, HG	Mixed urologic diseases and healthy	qMSP								91.38	93.33	0.923		2016
POU4F2, PCDH17, GDF15	Primary	Training	Sedimentation	58	90	Ta-T4, LG, HG	Mixed urologic diseases and healthy	qMSP								91.38	87.78	0.914		2016
POU4F2	Primary	Validation	Sedimentation	72	92	Ta-T4, LG, HG	Mixed urologic diseases and healthy	qMSP			86	92.5	86.1	91.49		88.89	93.48			2016
POU4F2, EOMES	Primary	Validation	Sedimentation	72	92	Ta-T4, LG, HG	Mixed urologic diseases and healthy	qMSP			92	92.5	91.67	92.55		95.83	88.04			2016
POU4F2, PCDH17	Primary	Validation	Sedimentation	72	92	Ta-T4, LG, HG	Mixed urologic diseases and healthy	qMSP			86	92.5	86.1	91.49		88.89	94.57			2016
POU4F2, PCDH17, GDF15	Primary	Validation	Sedimentation	72	92	Ta-T4, LG, HG	Mixed urologic diseases and healthy	qMSP			92	96.25	97.22	93.62		97.22	75.00			2016
HS3ST2	Primary		Filtration (11μm)	167	105	Ta-T1 (NMIBC), LG, HG	Patients with negative cystoscopy (hematuria)	qMSP								82.0	21.20			2016
SEPTIN9	Primary		Filtration (11μm)	167	105	Ta-T1 (NMIBC), LG, HG	Patients with negative cystoscopy (hematuria)	qMSP								90.4	67.6		[20]	2016
SLIT2	Primary		Filtration (11μm)	167	105	Ta-T1 (NMIBC), LG, HG	Patients with negative cystoscopy (hematuria)	qMSP								90.4	18.1			2016

HS3ST2, SEPTIN9	Primary		Filtration (11µm)	167	105	Ta-T1 (NMIBC), LG, HG	Patients with negative cystoscopy (hematuria)	qMSP									90.4	72.4			2016
HS3ST2, SLIT2	Primary		Filtration (11 µm)	167	105	Ta-T1 (NMIBC), LG, HG	Patients with negative cystoscopy (hematuria)	qMSP									90.4	34.3			2016
SEPTIN9, SLIT2	Primary		Filtration (11 µm)	167	105	Ta-T1 (NMIBC), LG, HG	Patients with negative cystoscopy (hematuria)	qMSP									91.0	71.4			2016
HS3ST2, SEPTIN9, SLIT2	Primary		Filtration (11 µm)	167	105	Ta-T1 (NMIBC), LG, HG	Patients with negative cystoscopy (hematuria)	qMSP	90.5	100	100	90 (other)		89	100		90.4	75.2			2016
HS3ST2, SLIT2, SEPTIN9	Recurrent		Filtration (11 µm)	72	86	Ta-T4, LG, HG	Ta-T4, LG, HG	qMSP	89.1	100	90.9	50 (other)		85.1	96						2016
TWIST1, NID2	Primary/Recurrent		Sedimentation	172 (37% hematuria and 63% NMIBC)		Ta-T1, LG, HG	Patients with negative cystoscopy (hematuria)	q-MSP											0.669	[63]	2017
CFTR, SALL3, TWIST1	Primary	Training	Sedimentation	111	57	Ta-T2, LG, HG	Mixed urologic diseases	Pyrosequencing						76.6	90.62		84.68	68.42	0.874	[28]	2018
CFTR, SALL3, TWIST1	Recurrent	Validation	Sedimentation	173	285	Ta-T1	Ta-T1	Pyrosequencing						90.62	88.31		89.6	30.53	0.741		2018
p14ARF	Primary		Sedimentation	113		≥T1, PUNLMP, grades 1–3	Healthy	MSP				57	27	18	48	24	32				2017
p16INK4a	Primary		Sedimentation	113		≥T1, PUNLMP, grades 1–3	Healthy	MSP				13	13	9	17	12	12			[19]	2017
RASSF1A	Primary		Sedimentation	113		≥T1, PUNLMP,	Healthy	MSP				57	44	41	56	35	46				2017

					grades 1–3											
DAPK1	Primary	Sedimentation	113		≥T1, PUNLMP, grades 1–3	Healthy	MSP	22	17	20	13	24	17			2017
APC	Primary	Sedimentation	113		≥T1, PUNLMP, grades 1–3	Healthy	MSP	66	43	43	56	35	46			2017
p14ARF, p16INK4A, RASSF1A, DAPK, APC	Primary	Sedimentation	113		≥T1, PUNLMP, grades 1–3	Healthy	MSP						91	-		2017
RARβ	Primary	Sedimentation	22	17	NMIBC-MIBC, grades 1–3	Healthy	MSP			69.2 (LG+HG)		66.7	68.2	76.4		2002
DAPK	Primary	Sedimentation	22	17	NMIBC-MIBC, grades 1–3	Healthy	MSP			38.4 (LG+HG)		55.5	45.5	100		2002
E-cad	Primary	Sedimentation	22	17	NMIBC-MIBC, grades 1–3	Healthy	MSP			53.8 (LG+HG)		66.7	59.1	100	[21]	2002
p16	Primary	Sedimentation	22	17	NMIBC-MIBC, grades 1–3	Healthy	MSP			7.6		22.2	13.6	100		2002
RARβ, DAPK, E-cad, p16	Primary	Sedimentation	22	17	NMIBC-MIBC, grades 1–3	Healthy	MSP			84.6 (LG+HG)		100	90.9	76.4		2002
APC, RASSF1A, p14ARF	Primary		45	38	Ta-T4, grades 1–3	Mixed urologic diseases and healthy	MSP						87	100	[42]	2004
BCL2	Primary	Sedimentation	37	20	Ta-T4	Healthy	qMSP						64.9	NA		2004
TERT	Primary	Sedimentation	37	20	Ta-T4	Healthy	qMSP						51.4	NA		2004
DAPK	Primary	Sedimentation	37	20	Ta-T4	Healthy	qMSP						21.6	NA	[43]	2004
BCL2, TERT, DAPK	Primary	Sedimentation	37	20	Ta-T4	Healthy	qMSP						78.4	78.4		2004
LAMA3	Primary	Sedimentation	71		MIBC, NMIBC, LG, HG		MSP						39	NA	[64]	2004
LAMB3	Primary	Sedimentation	71		MIBC, NMIBC		MSP						19	NA		2004

					, LG, HG												
LAMC2	Primary	Sedimentation	71		MIBC, NMIBC, LG, HG		MSP						15	NA			2004
LAMA3, LAMB3, LAMC2	Primary	Sedimentation	71		MIBC, NMIBC, LG, HG		MSP						49	NA			2004
sFRP-1	Primary	Sedimentation	24	20	MIBC, NMIBC, grades 1–3	Healthy	MSP	90	35.7	63.6	75	20	58.3	95			2006
sFRP-2	Primary	Sedimentation	24	20	MIBC, NMIBC, grades 1–3	Healthy	MSP	90	57.1	81.8	62.5	60	70.8	90			2006
sFRP-4	Primary	Sedimentation	24	20	MIBC, NMIBC, grades 1–3	Healthy	MSP	70	42.9	54.5	62.5	40	54.2	90			2006
sFRP-5	Primary	Sedimentation	24	20	MIBC, NMIBC, grades 1–3	Healthy	MSP	90	64.3	81.8	75	60	75.0	95		[65]	2006
Wif-1	Primary	Sedimentation	24	20	MIBC, NMIBC, grades 1–3	Healthy	MSP	70	35.7	45.5	62.5	40	58.3	95			2006
Dkk-3	Primary	Sedimentation	24	20	MIBC, NMIBC, grades 1–3	Healthy	MSP	90	35.7	63.6	75	20	50.0	95			2006
sFRP-1, sFRP-2, sFRP-4, sFRP-5,Wif-1, Dkk-3	Primary	Sedimentation	24	20	MIBC, NMIBC, grades 1–3	Healthy	MSP	83.3	45.2	65.2	68.8	33.3	61.1	93.3	0.763		2006
CDKN2A	Primary	Sedimentation	175	69	Ta-T4, grades 1–3	Mixed urologic diseases, hematuria	qMSP						45	NA			2006
ARF	Primary	Sedimentation	175	69	Ta-T4, grades 1–3	Mixed urologic diseases, hematuria	qMSP						28	NA			2006
MGMT	Primary	Sedimentation	175	69	Ta-T4, grades 1–3	Mixed urologic diseases, hematuria	qMSP						35	NA		[66]	2006
GSTP1	Primary	Sedimentation	175	69	Ta-T4, grades 1–3	Mixed urologic diseases, hematuria	qMSP						43	NA			2006
CDKN2A, ARF, MGMT, GSTP1	Primary	Sedimentation	175	69	Ta-T4, grades 1–3	Mixed urologic	qMSP	85	75				69	100			2006

						diseases, hematuria													
RASSF1A, E-cad, APC	Primary	Sedimentation	35	(>70 y)=35, (<40 Y)=34	Ta-T4	Hematuria or lower urinary tract symptoms	qMSP								69	60			2006
RASSF1A	Primary	Sedimentation	35	(>70 y)=35/ (<40 Y)=34	Ta-T4	Hematuria or lower urinary tract symptoms	qMSP								51	57/74			2006
E-cad	Primary	Sedimentation	35	(>70 y)=35/ (<40 Y)=34	Ta-T4	Hematuria or lower urinary tract symptoms	qMSP								31	49/62			2006
RAR-B	Primary	Sedimentation	35	(>70 y)=35/ (<40 Y)=34	Ta-T4	Hematuria or lower urinary tract symptoms	qMSP								3	100/100			2006
pl4	Primary	Sedimentation	35	(>70 y)=35/ (<40 Y)=34	Ta-T4	Hematuria or lower urinary tract symptoms	qMSP								0	100/100		[67]	2006
pl6	Primary	Sedimentation	35	(>70 y)=35/ (<40 Y)=34	Ta-T4	Hematuria or lower urinary tract symptoms	qMSP								0	100/100			2006
DAPK	Primary	Sedimentation	35	(>70 y)=35/ (<40 Y)=34	Ta-T4	Hematuria or lower urinary tract symptoms	qMSP								6	97/94			2006
GSTP1	Primary	Sedimentation	35	(>70 y)=35/ (<40 Y)=34	Ta-T4	Hematuria or lower urinary tract symptoms	qMSP								3	100/100			2006
APC	Primary	Sedimentation	35	(>70 y)=35/ (<40 Y)=34	Ta-T4	Hematuria or lower urinary tract symptoms	qMSP								40	80/88			2006
HOXA9, PCDH17, POU4F2, ONECUT2	Primary	Sedimentation	Total= 111		Ta-T4, LG, HG PUNLMP	Mixed urologic diseases, hematuria	qMSP	85.5	96.3 (CIS+ T1-T4)			91.5	88.4	33.3	90.5	73.2	0.871		2018
HOXA9	Primary	Sedimentation	Total= 111		Ta-T4, LG, HG PUNLMP	Mixed urologic diseases, hematuria	qMSP								50.9	93.1		**[22]**	2018
PCDH17	Primary	Sedimentation	Total= 111		Ta-T4, LG, HG	Mixed urologic	qMSP								47.2	79.3			2018

						PUNL MP	diseases, hematuria													
POU4F2	Primary		Sedimentation	Total= 111		Ta-T4, LG, HG PUNL MP	Mixed urologic diseases, hematuria	qMSP								77.4	79.3			2018
ONECUT2	Primary		Sedimentation	Total= 111		Ta-T4, LG, HG PUNL MP	Mixed urologic diseases, hematuria	qMSP								56.6	91.4			2018
CDH1	Primary		Sedimentation	48	35	Ta-T3, LG, HG	Healthy	MSP			61.5	68.8	66.7			67.4	93.9			2018
p14ARF	Primary		Sedimentation	48	35	Ta-T3, LG, HG	Healthy	MSP			76.9	68.8	75			72.1	63.6		[68]	2018
CDH1, p14ARF	Primary		Sedimentation	48	35	Ta-T3, LG, HG	Healthy	MSP					83.3			86.0	97.0			2018
ABCC6	Primary		Sedimentation	109	256	Ta-T4, LG, HG	Mixed urologic diseases, negative findings	MSP								52	53	0.527		2018
BRCA1	Primary		Sedimentation	109	256	Ta-T4, LG, HG	Mixed urologic diseases, negative findings	MSP								33	58	0.453		2018
GDF15	Primary		Sedimentation	109	256	Ta-T4, LG, HG	Mixed urologic diseases, negative findings	MSP								81	10	0.452		2018
HSPA2	Primary		Sedimentation	109	256	Ta-T4, LG, HG	Mixed urologic diseases, negative findings	MSP								73	36	0.548	[69]	2018
RASSF1A	Primary		Sedimentation	109	256	Ta-T4, LG, HG	Mixed urologic diseases, negative findings	MSP								51	81	0.624		2018
SALL3	Primary		Sedimentation	109	256	Ta-T4, LG, HG	Mixed urologic diseases, negative findings	MSP								28	86	0.540		2018
THBS1	Primary		Sedimentation	109	256	Ta-T4, LG, HG	Mixed urologic diseases, negative findings	MSP								84	25	0.546		2018
TMEFF2	Primary		Sedimentation	109	256	Ta-T4, LG, HG	Mixed urologic diseases, negative findings	MSP								68	40	0.539		2018

CDH1	Primary	Sedimentation	109	256	Ta-T4, LG, HG	Mixed urologic diseases, negative findings	MSP					13	98	0.555		2018
VIM	Primary	Sedimentation	109	256	Ta-T4, LG, HG	Mixed urologic diseases, negative findings	MSP					70	61	0.654		2018
VIM, RASSF1A	Primary	Sedimentation	109	256	Ta-T4, LG, HG	Mixed urologic diseases, negative findings	MSP					73	63	0.718		2018
VIM, RASSF1A, GDF15	Primary	Sedimentation	109	256	Ta-T4, LG, HG	Mixed urologic diseases, negative findings	MSP					45	91	0.746		2018
VIM, RASSF1A, GDF15, TMEFF2	Primary	Sedimentation	109	256	Ta-T4, LG, HG	Mixed urologic diseases, negative findings	MSP					82	53	0.760		2018
RBBP8	Primary	Sedimentation	22	10	Ta-T4, LG, HG	Healthy donors	MSP					50	100			2018
RBBP8	Primary	Sedimentation	52	51	Ta-T4, LG, HG	Mixed urologic diseases, disease free	Pyrosequencing					51.9	90.9	0.730	[70]	2018
ONECUT2	Primary	Filtration 8µm	99	376	Ta-T4, LG, HG, PUNLMP	Macroscopic hematuria, no malignancy	qMSP	58.7	100	93.9	92.3	77.8	93.9			2016
VIM	Primary	Filtration 8µm	99	376	Ta-T4, LG, HG, PUNLMP	Macroscopic hematuria, no malignancy	qMSP	69.6	71.4	81.8	84.6	75.8	95.7		[23]	2016
SALL3	Primary	Filtration 8µm	99	376	Ta-T4, LG, HG, PUNLMP	Macroscopic hematuria, no malignancy	qMSP	47.8	85.7	84.8	84.6	67.7	96.5			2016
CCNA1	Primary	Filtration 8µm	99	376	Ta-T4, LG, HG, PUNLMP	Macroscopic hematuria, no malignancy	qMSP	54.3	71.4	78.8	76.9	66.7	96.5			2016

BCL2	Primary		Filtration 8μm	99	376	Ta-T4, LG, HG, PUNLMP	Macroscopic hematuria, no malignancy	qMSP	54.3	71.4	69.7	69.2			62.6	97.9		2016
EOMES	Primary		Filtration 8μm	99	376	Ta-T4, LG, HG, PUNLMP	Macroscopic hematuria, no malignancy	qMSP	39.1	42.9	60.6	30.8			45.5	96.8		2016
ONECUT2, VIM, SALL3, CCNA1, BCL2, EOMES	Primary		Filtration 8μm	99	376	Ta-T4, LG, HG, PUNLMP	Macroscopic hematuria, no malignancy	qMSP							89.9	88.6		2016
IRF8	Primary		Sedimentation	26	19	Ta-T4, LG, HG	Noncancer, not specified	qMSP					50	68.8	61.5	94.7		2015
SFRP1	Primary		Sedimentation	26	19	Ta-T4, LG, HG	Noncancer, not specified	qMSP					60	43.8	50	94.7		2015
ZNF671	Primary		Sedimentation	26	19	Ta-T4, LG, HG	Noncancer, not specified	qMSP					40	68.8	57.7	89.5		2015
IRF8, SFRP1	Primary		Sedimentation	26	19	Ta-T4, LG, HG	Noncancer, not specified	qMSP					90	87.5	88.4	89.5		2015
ZNF671, IRF8	Primary		Sedimentation	26	19	Ta-T4, LG, HG	Noncancer, not specified	qMSP					60	94.1	80.8	84.2	[12]	2015
ZNF671, SFRP1	Primary		Sedimentation	26	19	Ta-T4, LG, HG	Noncancer, not specified	qMSP					80	87.5	84.6	89.5		2015
ZNF671, SFRP1, IRF8	Primary		Sedimentation	26	19	Ta-T4, LG, HG	Noncancer, not specified	qMSP					90	100	96.2	84.2		2015
ZNF671	Primary	Training	Sedimentation	69	28	Ta-T4, LG, HG	Noncancer, not specified	qMSP					45	40	42	92.8		2015
ZNF671	Primary	Validation	Sedimentation	33	28	Ta-T4, LG, HG	Noncancer, not specified	qMSP					33	52	48	89		2015
BCL2	Primary							qMSP							NA	NA		2015
EOMES	Primary		Filtration (8μm)	33	26	Ta-T2, LG, HG, PUNLMP	Mixed urologic diseases, negative findings	qMSP							29	NA	[14]	2015
VIM	Primary		Filtration (8μm)	33	26	Ta-T2, LG, HG, PUNLMP	Mixed urologic diseases, negative findings	qMSP							81	NA		2015

SALL3	Primary	Filtration (8µm)	33	26	Ta-T2, LG, HG, PUNLMP	Mixed urologic diseases, negative findings	qMSP									NA	NA			2015
CCNA1	Primary	Filtration (8µm)	33	26	Ta-T2, LG, HG, PUNLMP	Mixed urologic diseases, negative findings	qMSP									NA	NA			2015
HOXA9	Primary	Filtration (8µm)	33	26	Ta-T2, LG, HG, PUNLMP	Mixed urologic diseases, negative findings	qMSP									74	NA			2015
BCL2, EOMES, VIM, SALL3, CCNA1, HOXA9, POU4F2	Primary	Filtration (8µm)	33	26	Ta-T2, LG, HG, PUNLMP	Mixed urologic diseases, negative findings	qMSP									94	NA			2015
TWIST1, NID2	Primary	NA	222 (25% BC)		Ta-T1, LG, HG	Negative findings	qMSP									75	86	0.656	[71]	2015
CCND2	Primary	Sedimentation	148	56	Stage 1–3, LG, HG	Normal controls, not specified	qMSP				38.1, Invasive (stage 2, 3)	9.3 Noninvasive (stage 1)	34.6	12.5		25.6	100			2014
CCNA1	Primary	Sedimentation	73	60	Stage 1–3, LG, HG	Normal controls, not specified	qMSP				69.4 Invasive (stage 2, 3)	56.3 Noninvasive (stage 1)	67.3	50		68.4	83.4		[72]	2014
CALCA	Primary	Sedimentation	148	56	Stage 1–3, LG, HG	Normal controls, not specified	qMSP				72.8 Invasive (stage 2, 3)	53.1 Noninvasive (stage 1)	75.2	33.3		63.5	71.5			2014
VGF	Primary	Sedimentation	20	20			qMSP									40	95		[73]	2014
CCNA1, EOMES, HOXA9, POU4F2, SALL3, VIM2, BCL2	Primary	Sedimentation	189		≥Ta		qMSP	75 (LG Ta) 77 (HG Ta)	92	90	89					80			[46]	2014
CCNA1, EOMES, HOXA9, POU4F2, SALL3, VIM2, BCL2	Primary	Filtered cells (8µm)	187				qMSP	84 (LG Ta) 81 (HG Ta)	96	93	95					87			[46]	2014

TWIST1, NID2	Primary		Sedimentation	24	87												38	86	0.71	[74]	2014
OTX	Primary		Sedimentation	54	115	≥Ta, grade 1–3	Hematuria with nonmalignant causes	qMSP (SNUPE)											0.69		2013
ONECUT	Primary		Sedimentation	54	115	≥Ta, grade 1–3	Hematuria with nonmalignant causes	qMSP (SNUPE)											0.78		2013
OSR	Primary		Sedimentation	54	115	≥Ta, grade 1–3	Hematuria with nonmalignant causes	qMSP (SNUPE)											0.75	[75]	2013
SIM	Primary		Sedimentation	54	115	≥Ta, grade 1–3	Hematuria with nonmalignant causes	qMSP (SNUPE)											0.60		2013
MEIS	Primary		Sedimentation	54	115	≥Ta, grade 1–3	Hematuria with nonmalignant causes	qMSP (SNUPE)											0.71		2013
OTX1	Primary	Training	Sedimentation	101	70	Recurrent Ta-T2, grade 1–3	Healthy	BS-SNaPshot	60		77	86			81	48	65	Fixed=90%	0.805		2013
MEIS1	Primary	Training	Sedimentation	101	70	Recurrent Ta-T2, grade 1–3	Healthy		44		62	50		48	59	30	46	Fixed=90%	0.749		2013
ONECUT2	Primary	Training	Sedimentation	101	70	Recurrent Ta-T2, grade 1–3	Healthy		49		77	50		46	70	43	52	Fixed=90%	0.737		2013
SIM2	Primary	Training	Sedimentation	101	70	Recurrent Ta-T2, grade 1–3	Healthy		40		85	71		46	78	22	49	Fixed=90%	0.753	[30]	2013
FOXA1	Primary	Training	Sedimentation	101	70	Recurrent Ta-T2, grade 1–3	Healthy		33		62	43		43	59	4	38	Fixed=90%	0.659		2013
ZNF503	Primary	Training	Sedimentation	101	70	Recurrent Ta-T2, grade 1–3	Healthy		47		77	64		50	70	35	52	Fixed=90%	0.784		2013

HOXA9	Primary	Training	Sedimentation	101	70	Recurrent Ta-T2, grade 1–3	Healthy		60	77	64	61	78	52	62	Fixed=90%	0.829		2013
OSR1	Primary	Training	Sedimentation	101	70	Recurrent Ta-T2, grade 1–3	Healthy		37	69	57	37	74	17	44	Fixed=90%	0.705		2013
OTX1, ONECUT2, OSR1	Primary	Training	Sedimentation	101	70	Recurrent Ta-T2, grade 1–3	Healthy		64	77	86	65	81	57	68	Fixed=90%	0.801		2013
OTX1, ONECUT2, OSR1	Recurrent	Validation	Sedimentation	95	40	Pre-tur, NMIBC, grade 1–3, (recurrence)	No recurrence								74	Fixed=90%	0.864		2013
SOX1	Primary		Sedimentation	73	18	T1-T4, grade 1–3	Healthy	Pyrosequencing							41.54	100	0.74		2013
TJP2	Primary		Sedimentation	73	18	T1-T4, grade 1–3	Healthy	Pyrosequencing							92.54	56.25	0.79		2013
MYOD	Primary		Sedimentation	73	18	T1-T4, grade 1–3	Healthy	Pyrosequencing							86.79	87.50	0.93		2013
HOXA9_1	Primary		Sedimentation	73	18	T1-T4, grade 1–3	Healthy	Pyrosequencing							86.23	88.89	0.92		2013
HOXA9_2	Primary		Sedimentation	73	18	T1-T4, grade 1–3	Healthy	Pyrosequencing							88.57	61.54	0.81		2013
VAMP8 (hypomethyated)	Primary		Sedimentation	73	18	T1-T4, grade 1–3	Healthy	Pyrosequencing							97.06	40.0	0.72	[10]	2013
CASP8 (hypomethyated)	Primary		Sedimentation	73	18	T1-T4, grade 1–3	Healthy	Pyrosequencing							73.61	76.92	0.82		2013
SPP1 (hypomethyated)	Primary		Sedimentation	73	18	T1-T4, grade 1–3	Healthy	Pyrosequencing							85.94	75.0	0.79		2013
SOX1, TJP2, MYOD, HOXA9_1, HOXA9_2, VAMP8, CASP8, SPP1, IFNG, CAPG, HLADPA1, RIPK3 (positive when six or more are present)	Primary		Sedimentation	73	18	Ta-T4, grade 1–3	Healthy	Pyrosequencing							100	100			2013
TWIST1	Primary		Sedimentation	24	15	Ta-T3, LG, HG	Mixed urologic diseases,	MSP							87.5	93.3		[13]	2013

							and healthy													
NID2	Primary		Sedimentation	24	15	Ta-T3, LG, HG	Mixed urologic diseases, and healthy	MSP								95.8	100			2013
TWIST1, NID2	Primary		Sedimentation	24	15	Ta-T3, LG, HG	Mixed urologic diseases, and healthy	MSP								95.8	93.3			2013
PRDM2	Primary	Training	Sedimentation	70	50	≥Ta, LG, HG	Mixed urologic diseases, and healthy	MS-MLPA			10.5	21.6	23.5	13.9		18.6	72	0.522		2013
RUNX3	Primary	Training	Sedimentation	70	50	≥Ta, LG, HG	Mixed urologic diseases, and healthy	MS-MLPA			31.6	29.4	32.3	27.8		30	86	0.613		2013
RARB	Primary	Training	Sedimentation	70	50	≥Ta, LG, HG	Mixed urologic diseases, and healthy	MS-MLPA			10.5	29.4	35.3	13.9		24.3	70	0.496		2013
HLTF-1	Primary	Training	Sedimentation	70	50	≥Ta, LG, HG	Mixed urologic diseases, and healthy	MS-MLPA			0.0	15.7	20.6	2.8		11.4	80	0.453		2013
HLTF-2	Primary	Training	Sedimentation	70	50	≥Ta, LG, HG	Mixed urologic diseases, and healthy	MS-MLPA			0.0	9.8	8.8	5.5		7.1	82	0.44	[76]	2013
SCGB3A1-1	Primary	Training	Sedimentation	70	50	≥Ta, LG, HG	Mixed urologic diseases, and healthy	MS-MLPA			21.0	35.3	41.2	22.2		31.4	68	0.526		2013
SCGB3A1-2	Primary	Training	Sedimentation	70	50	≥Ta, LG, HG	Mixed urologic diseases, and healthy	MS-MLPA			0.0	15.7	17.6	5.5		11.4	76	0.429		2013
ID4-1	Primary	Training	Sedimentation	70	50	≥Ta, LG, HG	Mixed urologic diseases, and healthy	MS-MLPA			10.5	37.2	38.2	22.2		30	82	0.609		2013
ID4-2	Primary	Training	Sedimentation	70	50	≥Ta, LG, HG	Mixed urologic diseases, and healthy	MS-MLPA			0.0	13.7	14.7	5.5		10	80	0.51		2013

TWIST1	Primary	Training	Sedimentation	70	50	≥Ta, LG, HG	Mixed urologic diseases, and healthy	MS-MLPA	21.0	19.6	14.7	25.0	20	74	0.56	2013
SFRP4-1	Primary	Training	Sedimentation	70	50	≥Ta, LG, HG	Mixed urologic diseases, and healthy	MS-MLPA	15.8	21.6	23.5	16.7	20	74	0.489	2013
SFRP4-2	Primary	Training	Sedimentation	70	50	≥Ta, LG, HG	Mixed urologic diseases, and healthy	MS-MLPA	10.5	19.6	20.6	13.9	17.1	78	0.48	2013
DLC1-1	Primary	Training	Sedimentation	70	50	≥Ta, LG, HG	Mixed urologic diseases, and healthy	MS-MLPA	10.5	17.6	17.6	13.9	15.7	74	0.511	2013
DLC1-2	Primary	Training	Sedimentation	70	50	≥Ta, LG, HG	Mixed urologic diseases, and healthy	MS-MLPA	5.3	21.6	23.5	11.1	17.1	80	0.538	2013
SFRP5-1	Primary	Training	Sedimentation	70	50	≥Ta, LG, HG	Mixed urologic diseases, and healthy	MS-MLPA	10.5	17.6	20.6	11.1	15.7	80	0.499	2013
SFRP5-2	Primary	Training	Sedimentation	70	50	≥Ta, LG, HG	Mixed urologic diseases, and healthy	MS-MLPA	5.3	13.7	14.7	8.3	11.4	88	0.546	2013
BNIP3	Primary	Training	Sedimentation	70	50	≥Ta, LG, HG	Mixed urologic diseases, and healthy	MS-MLPA	15.8	33.3	35.3	22.2	28.6	70	0.506	2013
H2AFX-1	Primary	Training	Sedimentation	70	50	≥Ta, LG, HG	Mixed urologic diseases, and healthy	MS-MLPA	5.3	19.6	26.5	5.5	15.7	84	0.551	2013
H2AFX-2	Primary	Training	Sedimentation	70	50	≥Ta, LG, HG	Mixed urologic diseases, and healthy	MS-MLPA	0.0	11.8	14.7	2.8	8.6	90	0.548	2013
CCND2-1	Primary	Training	Sedimentation	70	50	≥Ta, LG, HG	Mixed urologic diseases, and healthy	MS-MLPA	21.0	25.5	20.6	25.0	24.3	82	0.589	2013
CCND2-2	Primary	Training	Sedimentation	70	50	≥Ta, LG, HG	Mixed urologic	MS-MLPA	31.6	43.1	47.1	33.3	40	66	0.568	2013

							diseases, and healthy												
CACNA1G	Primary	Training	Sedimentation	70	50	≥Ta, LG, HG	Mixed urologic diseases, and healthy	MS-MLPA		10.5	13.7	14.7	11.1		12.9	78	0.491		2013
TGIF	Primary	Training	Sedimentation	70	50	≥Ta, LG, HG	Mixed urologic diseases, and healthy	MS-MLPA		5.3	17.6	20.6	8.3		14.3	76	0.416		2013
BCL2	Primary	Training	Sedimentation	70	50	≥Ta, LG, HG	Mixed urologic diseases, and healthy	MS-MLPA		0.0	11.8	11.8	5.5		8.6	86	0.503		2013
CACNA1A	Primary	Training	Sedimentation	70	50	≥Ta, LG, HG	Mixed urologic diseases, and healthy	MS-MLPA		46.1	15.7	14.7	13.9		14.3	82	0.562		2013
TIMP3-1	Primary	Training	Sedimentation	70	50	≥Ta, LG, HG	Mixed urologic diseases, and healthy	MS-MLPA		5.3	11.8	14.7	5.5		10	84	0.511		2013
TIMP3-2	Primary	Training	Sedimentation	70	50	≥Ta, LG, HG	Mixed urologic diseases, and healthy	MS-MLPA		10.5	13.7	14.7	11.1		12.9	80	0.481		2013
PRDM2	Primary	Validation	Sedimentation	100	28	≥Ta, LG, HG	Mixed urologic diseases, and healthy	MS-MLPA		42.3	28.4	23.9	38.9		32	71.4	0.565		2013
RUNX3	Primary	Validation	Sedimentation	100	28	≥Ta, LG, HG	Mixed urologic diseases, and healthy	MS-MLPA		46.1	33.8	26.1	46.3		37	82.1	0.655		2013
RARB	Primary	Validation	Sedimentation	100	28	≥Ta, LG, HG	Mixed urologic diseases, and healthy	MS-MLPA		19.2	14.8	17.4	14.8		16	85.7	0.521		2013
HLTF-1	Primary	Validation	Sedimentation	100	28	≥Ta, LG, HG	Mixed urologic diseases, and healthy	MS-MLPA		3.8	16.2	8.7	16.7		13	89.3	0.465		2013
HLTF-2	Primary	Validation	Sedimentation	100	28	≥Ta, LG, HG	Mixed urologic diseases,	MS-MLPA		7.7	12.2	4.3	16.7		11	92.9	0.511		2013

							and healthy									
SCGB3A1-1	Primary	Validation	Sedimentation	100	28	≥Ta, LG, HG	Mixed urologic diseases, and healthy	MS-MLPA	42.3	41.9	37	46.3	42	71.4	0.543	2013
SCGB3A1-2	Primary	Validation	Sedimentation	100	28	≥Ta, LG, HG	Mixed urologic diseases, and healthy	MS-MLPA	19.2	10.8	10.9	14.8	13	92.9	0.533	2013
ID4-1	Primary	Validation	Sedimentation	100	28	≥Ta, LG, HG	Mixed urologic diseases, and healthy	MS-MLPA	26.9	28.4	26.1	29.6	28	71.4	0.539	2013
ID4-2	Primary	Validation	Sedimentation	100	28	≥Ta, LG, HG	Mixed urologic diseases, and healthy	MS-MLPA	3.8	21.6	19.6	14.8	17	82.1	0.535	2013
TWIST1	Primary	Validation	Sedimentation	100	28	≥Ta, LG, HG	Mixed urologic diseases, and healthy	MS-MLPA	30.8	20.3	19.6	25.9	23	82.1	0.538	2013
SFRP4-1	Primary	Validation	Sedimentation	100	28	≥Ta, LG, HG	Mixed urologic diseases, and healthy	MS-MLPA	3.8	14.9	15.2	9.3	12	89.3	0.532	2013
SFRP4-2	Primary	Validation	Sedimentation	100	28	≥Ta, LG, HG	Mixed urologic diseases, and healthy	MS-MLPA	7.7	13.5	15.2	9.3	12	92.9	0.562	2013
DLC1-1	Primary	Validation	Sedimentation	100	28	≥Ta, LG, HG	Mixed urologic diseases, and healthy	MS-MLPA	30.8	18.9	19.6	24.1	22	82.1	0.533	2013
DLC1-2	Primary	Validation	Sedimentation	100	28	≥Ta, LG, HG	Mixed urologic diseases, and healthy	MS-MLPA	30.8	13.5	17.4	18.5	18	92.9	0.594	2013
SFRP5-1	Primary	Validation	Sedimentation	100	28	≥Ta, LG, HG	Mixed urologic diseases, and healthy	MS-MLPA	23.1	17.6	13	24.1	19	92.9	0.578	2013
SFRP5-2	Primary	Validation	Sedimentation	100	28	≥Ta, LG, HG	Mixed urologic diseases, and healthy	MS-MLPA	7.7	10.8	8.7	11.1	10	89.3	0.556	2013

BNIP3	Primary	Validation	Sedimentation	100	28	≥Ta, LG, HG	Mixed urologic diseases, and healthy	MS-MLPA	34.6	41.9	37	42.6	40	57.1	0.532	2013
H2AFX-1	Primary	Validation	Sedimentation	100	28	≥Ta, LG, HG	Mixed urologic diseases, and healthy	MS-MLPA	11.5	21.6	19.6	18.5	19	78.6	0.469	2013
H2AFX-2	Primary	Validation	Sedimentation	100	28	≥Ta, LG, HG	Mixed urologic diseases, and healthy	MS-MLPA	0	10.8	10.9	5.5	8	96.4	0.493	2013
CCND2-1	Primary	Validation	Sedimentation	100	28	≥Ta, LG, HG	Mixed urologic diseases, and healthy	MS-MLPA	15.4	27	19.6	27.8	24	92.9	0.598	2013
CCND2-2	Primary	Validation	Sedimentation	100	28	≥Ta, LG, HG	Mixed urologic diseases, and healthy	MS-MLPA	46.1	47.3	43.5	50	47	64.3	0.544	2013
CACNA1G	Primary	Validation	Sedimentation	100	28	≥Ta, LG, HG	Mixed urologic diseases, and healthy	MS-MLPA	7.7	20.3	13	20.4	17	92.9	0.608	2013
TGIF	Primary	Validation	Sedimentation	100	28	≥Ta, LG, HG	Mixed urologic diseases, and healthy	MS-MLPA	11.5	5.4	6.5	7.4	7	85.7	0.48	2013
BCL2	Primary	Validation	Sedimentation	100	28	≥Ta, LG, HG	Mixed urologic diseases, and healthy	MS-MLPA	3.8	12.2	8.7	11.1	10	89.3	0.508	2013
CACNA1A	Primary	Validation	Sedimentation	100	28	≥Ta, LG, HG	Mixed urologic diseases, and healthy	MS-MLPA	7.7	18.9	17.4	14.8	16	92.9	0.63	2013
TIMP3-1	Primary	Validation	Sedimentation	100	28	≥Ta, LG, HG	Mixed urologic diseases, and healthy	MS-MLPA	3.8	14.9	13	11.1	12	92.9	0.546	2013
TIMP3-2	Primary	Validation	Sedimentation	100	28	≥Ta, LG, HG	Mixed urologic diseases, and healthy	MS-MLPA	0	14.9	13	9.3	11	89.3	0.544	2013

Panel consisting of 41 sequences	Recurrent	Sedimentation	136 G1/G2 NMIBC		≥Ta, LG, HG	Mixed urologic diseases, and healthy	MS-MLPA	68		89	50		74 (grade 2)	82 (grade 3)	57 (grade 1), 20 (grade 0)				[31]	2013
BCL2, CDKN2A, NID2	Primary	Sedimentation	42	22	Ta-T3, LG, HG	Mixed urologic diseases, and healthy	nested MSP	61.1		92.3	100		83	94.4	54.5	80.95	86.36		[77]	2012
BCL2	Primary	Sedimentation	42	22	Ta-T3, LG, HG	Mixed urologic diseases, and healthy	nested MSP	38.8		69.2	100		66.7	83.3	27.3	61.90	100			2012
CDKN2A	Primary	Sedimentation	42	22	Ta-T3, LG, HG	Mixed urologic diseases, and healthy	nested MSP	11.1		7.7	0		0	5.6	18	7.14	95.45			2012
NID2	Primary	Sedimentation	42	22	Ta-T3, LG, HG	Mixed urologic diseases, and healthy	nested MSP	38.8		76.9	88.9		66.7	77.8	27.3	61.90	90.91			2012
EOMES	Primary	Sedimentation	184	35	Ta-T1, grade 1–3	Mixed urologic diseases	qMSP	86	100	92			85	95	65	88	97	0.96	[25]	2012
HOXA9	Primary	Sedimentation	184	35	Ta-T1, grade 1–3	Mixed urologic diseases	qMSP	80	50	87			80	83	80	82	100	0.91		2012
POU4F2	Primary	Sedimentation	184	35	Ta-T1, grade 1–3	Mixed urologic diseases	qMSP	83	100	88			82	91	59	85	94	0.94		2012
TWIST1	Primary	Sedimentation	184	35	Ta-T1, grade 1–3	Mixed urologic diseases	qMSP	85	100	96			90	89	71	88	100	0.94		2012
VIM	Primary	Sedimentation	184	35	Ta-T1, grade 1–3	Mixed urologic diseases	qMSP	89	100	88			86	94	75	89	100	0.97		2012
ZNF154	Primary	Sedimentation	184	35	Ta-T1, grade 1–3	Mixed urologic diseases	qMSP	84	100	94			77	98	71	87	100	0.95		2012
EOMES	Recurrent	Sedimentation	139	67	Ta-T1, grade 1–3	Mixed urologic diseases	qMSP	91	100	96	100		96	92	88	94	55	0.85		2012
HOXA9	Recurrent	Sedimentation	139	67	Ta-T1, grade 1–3	Mixed urologic diseases	qMSP	93	100	92	90		93	92	91	93	55	0.78		2012
POU4F2	Recurrent	Sedimentation	139	67	Ta-T1, grade 1–3	Mixed urologic diseases	qMSP	85	67	93	90		89	85	81	88	64	0.80		2012
TWIST1	Recurrent	Sedimentation	139	67	Ta-T1, grade 1–3	Mixed urologic diseases	qMSP	87	75	93	100		88	91	82	90	43	0.76		2012

VIM	Recurrent		Sedimentation	139	67	Ta-T1, grade 1–3	Mixed urologic diseases	qMSP	89	75	93	92		92	88	89	90	59	0.78	[78]	2012
ZNF154	Recurrent		Sedimentation	139	67	Ta-T1, grade 1–3	Mixed urologic diseases	qMSP	95	100	93	83		94	92	100	94	67	0.83		2012
TWIST1	Primary	Training	Sedimentation	48	275	Not specified	Hematuria	qMSP									84				2012
NID2	Primary	Training	Sedimentation	48	275	Not specified	Hematuria	qMSP									33				2012
VAX1	Primary and Recurrent	Validation	Sedimentation	212	149/41	Stage 1–4, grade 1–3	Normal/Urinary Lesions	MSP									42.45	95.31/87.81	73.3/59.5	[79]	2012
KCNV1	Primary and Recurrent	Validation	Sedimentation	212	149/41	Stage 1–4, grade 1–3	Normal/Urinary Lesions	MSP									36.92	93.96/95.12	71.3/60.5		2012
ECEL1	Primary and Recurrent	Validation	Sedimentation	212	149/41	Stage 1–4, grade 1–3	Normal/Urinary Lesions	MSP									26.89	97.31/97.56	70.8/59.3		2012
TMEM26	Primary and Recurrent	Validation	Sedimentation	212	149/41	Stage 1–4, grade 1–3	Normal/Urinary Lesions	MSP									26.42	96.64/97.56	69.4/60.3		2012
PROX1	Primary and Recurrent	Validation	Sedimentation	212	149/41	Stage 1–4, grade 1–3	Normal/Urinary Lesions	MSP									24.53	98.66/100.0	71.1/59.1		2012
TAL1	Primary and Recurrent	Validation	Sedimentation	212	149/41	Stage 1–4, grade 1–3	Normal/Urinary Lesions	MSP									24.83	98.66/100.0	72.5/60.4		2012
SLC6A20	Primary and Recurrent	Validation	Sedimentation	212	149/41	Stage 1–4, grade 1–3	Normal/Urinary Lesions	MSP									15.57	97.89/100.0	69.7/59.3		2012
LMX1	Primary and Recurrent	Validation	Sedimentation	212	149/41	Stage 1–4, grade 1–3	Normal/Urinary Lesions	MSP									9.43	98.66/100.0	67.1/58.8		2012
CFTR	Primary	Validation	Sedimentation	212	149/41	Stage 1–4, grade 1–3	Normal/Urinary Lesions	MSP									52.35	96.64/97.56	77.4/63.8		2012
VAX1, KCNV1, TAL1, PPOX1	Primary	Validation	Sedimentation	212	149/41	Stage 1–4, grade 1–3	Normal/Urinary Lesions	MSP									76.89	88.59/85.36	82.8/84.3		2012
CFTR, VAX1, KCNV1, TAL1, PPOX1	Primary	Validation	Sedimentation	212	149/41	Stage 1–4, grade 1–3	Normal/Urinary Lesions	MSP									88.68	87.25/90.0	89.9/90.0		2012

RAR-β2	Primary	Sedimentation	100	51	Stage 1–4, LG, HG	Not Specified	Methylation specific nested PCR				77.8 (stage III,,IV)	62.2 (stage I,II)	59.2	83.3		65	69.7		[80]	2012
TWIST1	Recurrent		48	275	Ta-T3, grade 1–3	No recurrence	MSP									75	69		[26]	2012
NID2	Recurrent		48	275	Ta-T3, grade 1–3	No recurrence	MSP									46	90			2012
PCDH17, TCF21	Primary	Sedimentation	50	48	Ta-T4, HG, LG	Healthy	qMSP									60	100			2011
PCDH17	Primary	Sedimentation	50	48	Ta-T4, HG, LG	Healthy	qMSP									50	100		[39]	2011
TCF21	Primary	Sedimentation	50	48	Ta-T4, HG, LG	Healthy	qMSP									52	100			2011
ZNF154	Primary	Sedimentation	110	57	Ta-T4, grade 1–4	Not specified	MS-HRM	84		100	100		95 (grade 2	97 g(grade 3–4)	67 (grade 1)	62	100	0.84		2011
POU4F2	Primary	Sedimentation	113	54	Ta-T4, grade 1–4	Not specified	MS-HRM	92		100	100		95 (grade 2)	97 (grade 3–4)	100 (grade 1)	66	100	0.88		2011
HOXA9	Primary	Sedimentation	107	48	Ta-T4, grade 1–4	Not specified	MS-HRM	83		100	87		88 (grade 2)	90 (grade 3–4)	100 (grade 1)	74	96	0.84		2011
EOMES	Primary	Sedimentation	101	40	Ta-T4, grade 1–4	Not specified	MS-HRM	68		93	87		79 (grade 2)	86 (grade 3–4)	50 (grade 1)	68	100	0.89		2011
CA3	Primary	Sedimentation			Ta-T4, grade 1–4	Not specified	MS-HRM	92		100	100		100 (grade 2)	100 (grade 3–4)	67 (grade 1)				[81]	2011
PCDHGA12	Primary	Sedimentation			Ta-T4, grade 1–4	Not specified	MS-HRM	92		93	100		95 (grade 2)	97 (grade 3–4)	83 (grade 1)					2011
ACOT11	Primary	Sedimentation			Ta-T4, grade 1–4	Not specified	MS-HRM	79		100	100		78 (grade 2)	97 (grade 3–4)	100 (grade 1)					2011
PTGDR	Primary	Sedimentation			Ta-T4, grade 1–4	Not specified	MS-HRM	44		80	67		58 (grade 2)	69 (grade 3–4)	33 (grade 1)					2011
ZNF154, POU4F2, HOXA9, EOMES	Primary	Sedimentation	112	52	Ta-T4, grade 1–4	Not specified	MS-HRM									84	96	0.90		2011
RARb2	Primary	Sedimentation	210	110	Stage 1–4, grade 1–3	Healthy/benign BC	MSP	70 (stage 1)	52.2. (stage 2)	53.3, (stage 3)	69.4, (stage 4)		58.2	52.6	83.7	62.8	87.9		[82]	2011
APC	Primary	Sedimentation	210	110	Stage 1–4, grade 1–3	Healthy/benign BC	MSP	58.4 (stage 1)	64.2. (stage) 2	73.3, (stage 3)	41.7, (stage 4)		59.1	63.2	55.8	59.5	98			2011

RARb2, APC	Primary	Sedimentation	210	110	Stage 1–4, grade 1–3	Healthy/benign BC	MSP			87.3	97.6			2011
DAPK	Primary/Recurrent	Sedimentation	30	19	≥Ta, grade 1–3	Noncancer controls, not further specified	qMSP	25.0 (srade 1)	27.7 (grade 2-3)	26.7	89.5		[83]	2011
IRF8	Primary/Recurrent	Sedimentation	30	19	≥Ta, grade 1–3	Noncancer controls, not further specified	qMSP	50.0 (grade 1)	61.1 (grade 2–3)	56.7	94.7			2011
p14	Primary/Recurrent	Sedimentation	30	19	≥Ta, grade 1–3	Noncancer controls, not further specified	qMSP	41.7 (grade 1)	16.7 (grade 2–3)	27.6	100			2011
RASSF1A	Primary/Recurrent	Sedimentation	30	19	≥Ta, grade 1–3	Noncancer controls, not further specified	qMSP	16.7 (grade 1)	38.9 (grade 2–3)	30.0	89			2011
SFRP1	Primary/Recurrent	Sedimentation	30	19	≥Ta, grade 1–3	Noncancer controls, not further specified	qMSP	50.0 (grade 1)	33.4 (grade 2–3)	41.4	100			2011
IRF8, p14, SFRP1	Primary/Recurrent	Sedimentation	30	19	≥Ta, grade 1–3	Noncancer controls, not further specified	qMSP	91.7 (grade 1)	83.3 (grade 2–3)	86.7	94.7			2011
MYO3A	Primary	Sedimentation	128	110	Ta-T4, grade 1–4	Benign urological disorders, healthy	qMSP			77.3	90.9	0.841	[84]	2011
CA10	Primary	Sedimentation	128	110	Ta-T4, grade 1–4	Benign urological disorders, healthy	qMSP			85.2	81.8	0.835		2011
NKX6-2	Primary	Sedimentation	128	110	Ta-T4, grade 1–4	Benign urological disorders, healthy	qMSP			88.3	76.4	0.823		2011
PENK	Primary	Sedimentation	128	110	Ta-T4, grade 1–4	Benign urological disorders, healthy	qMSP			81.3	79.1	0.802		2011
SOX11	Primary	Sedimentation	128	110	Ta-T4, grade 1–4	Benign urological disorders, healthy	qMSP			70.3	89.1	0.797		2011
DBC1	Primary	Sedimentation	128	110	Ta-T4, grade 1–4	Benign urological	qMSP			71.1	83.6	0.774		2011

						disorders, healthy						
NPTX2	Primary	Sedimentation	128	110	Ta-T4, grade 1–4	Benign urological disorders, healthy	qMSP	75.8	73.6	0.747		2011
A2BP1	Primary	Sedimentation	128	110	Ta-T4, grade 1–4	Benign urological disorders, healthy	qMSP	87.5	54.5	0.710		2011
MYO3A, CA10, NKX6-2, DBC1, SOX11	Primary	Sedimentation	128	110	Ta-T4, grade 1–4	Benign urological disorders, healthy	qMSP	85.2	94.5	0.939		2011
MYO3A, CA10, NKX6-2, DBC1, PENK	Primary	Sedimentation	128	110	Ta-T4, grade 1–4	Benign urological disorders, healthy	qMSP	85.2	94.5	0.939		2011
MYO3A, CA10, NKX6-2, SOX11	Primary	Sedimentation	128	110	Ta-T4, grade 1–4	Benign urological disorders, healthy	qMSP	81.3	97.3	0.939		2011
MYO3A, CA10, NKX6-2, DBC1	Primary	Sedimentation	128	110	Ta-T4, grade 1–4	Benign urological disorders, healthy	qMSP	81.3	97.3	0.939		2011
GDF15	Primary	Sedimentation	51	20	Not specified	Healthy	qMSP	47.1	100			2010
HSPA2	Primary	Sedimentation	51	20	Not specified	Healthy	qMSP	58.8	100			2010
TMEFF2	Primary	Sedimentation	51	20	Not specified	Healthy	qMSP	62.8	100			2010
VIM	Primary	Sedimentation	51	20	Not specified	Healthy	qMSP	78.4	100		[16]	2010
VIM, TMEFF2	Primary	Sedimentation	51	20	Not specified	Healthy	qMSP	82	100			2010
VIM, TMEFF2, GDF15	Primary	Sedimentation	51	20	Not specified	Healthy	qMSP	94	100			2010
VIM, TMEFF2, GDF15, HSPA2	Primary	Sedimentation	51	20	Not specified	Healthy	qMSP	94	100			2010
APC, RARB, RASSF1A, SFRP1, SFRP54, SFRP5, DBC1, SFRP2	Primary	Sedimentation	113	33	Ta-T3, grade 0-4	Mixed genitourinary disorders	qMSP	52	100		[85]	2011
SFRP1	Primary	Sedimentation	82	15	Stage pTa–IV	Mixed urologic diseases	MSP	36.6	93,33		[18]	2009

FANCF	Primary	Sedimentation	82	15	Stage pTa–IV	Mixed urologic diseases	MSP						13.4	100		2009
LOXL1	Primary	Sedimentation	82	15	Stage pTa–IV	Mixed urologic diseases	MSP						40.2	73,33		2009
p16INK4	Primary	Sedimentation	82	15	Stage pTa–IV	Mixed urologic diseases	MSP						22	86,67		2009
XAF1	Primary	Sedimentation	82	15	Stage pTa–IV	Mixed urologic diseases	MSP						70.7	33,33		2009
CDH1	Primary	Sedimentation	82	15	Stage pTa–IV	Mixed urologic diseases	MSP						22	80,00		2009
LOXL4	Primary	Sedimentation	82	15	Stage pTa–IV	Mixed urologic diseases	MSP						11	/86.7		2009
TIMP3	Primary	Sedimentation	82	15	Stage pTa–IV	Mixed urologic diseases	MSP						8.5	/86.7		2009
TIG1	Primary	Sedimentation	82	15	Stage pTa–IV	Mixed urologic diseases	MSP						3.7	93,33		2009
SOX9	Primary	Sedimentation	82	15	Stage pTa–IV	Mixed urologic diseases	MSP						3.7	86,67		2009
SALL3	Primary	Sedimentation	82	15	Stage pTa–IV	Mixed urologic diseases	MSP						58	100		2009
SALL3, CFTR, MT1A, HPP1, ABCC6, RASSF1A, CDH13, RPRM, MINT1, BRCA1	Primary	Sedimentation	82	15	Stage pTa–IV	Mixed urologic diseases	MSP						90.2	80.0		2009
SALL3, CFTR, MT1A, HPP1, ABCC6, RASSF1A, CDH13, RPRM, MINT1, BRCA1, SFRP1	Primary	Sedimentation	82	15	Stage pTa–IV	Mixed urologic diseases	MSP						91.5	73.3		2009
BCL2	Primary	Sedimentation	108	105	Ta-T1, LG, HG	Mixed urologic diseases, Healthy	qMSP	66	0	65	57	37	64.8	97.1		2011
hTERT	Primary	Sedimentation	108	105	Ta-T1, LG, HG	Mixed urologic diseases, Healthy	qMSP	66	50	38	23	70	41.7	99.1	[86]	2011
DAPK	Primary	Sedimentation	108	105	Ta-T1, LG, HG	Mixed urologic diseases, Healthy	qMSP	27	0	20	23	28	25	90.5		2011
BCL2, hTERT, DAPK	Primary	Sedimentation	108	105	Ta-T1, LG, HG	Mixed urologic diseases, Healthy	qMSP	79	50	80	71	91	78.7	89.5		2011

E-cad	Primary	Sedimentation	57	20	Ta-T4, LG, HG	Healthy	MSP				32	38	42	32	35	NA		[87]	2010
p16	Primary	Sedimentation	57	20	Ta-T4, LG, HG	Healthy	MSP				36	34	37	34	35				2010
p14	Primary	Sedimentation	57	20	Ta-T4, LG, HG	Healthy	MSP				24	41	42	29	33				2010
RASSF1A	Primary	Sedimentation	57	20	Ta-T4, LG, HG	Healthy	MSP				64	66	63	66	65				2010
p14, RASSF1A	Primary	Sedimentation	57	20	Ta-T4, LG, HG	Healthy	MSP								75				2010
E-cad, p14, RASSF1A	Primary	Sedimentation	57	20	Ta-T4, LG, HG	Healthy	MSP				75	85	85	79	83				2010
RASSF1A	Recurrent	Sedimentation	15	25	Ta-T1	No recurrence	qMSP								50	32		[24]	2008
ECAD	Recurrent	Sedimentation	15	25	Ta-T1	No recurrence	qMSP								7	84			2008
APC	Recurrent	Sedimentation	15	25	Ta-T1	No recurrence	qMSP								27	80			2008
DAPK	Recurrent	Sedimentation	15	25	Ta-T1	No recurrence	qMSP								0	96			2008
MGMT	Recurrent	Sedimentation	15	25	Ta-T1	No recurrence	qMSP								20	92			2008
BCL2	Recurrent	Sedimentation	15	25	Ta-T1	No recurrence	qMSP								13	96			2008
TERT	Recurrent	Sedimentation	15	25	Ta-T1	No recurrence	qMSP								13	100			2008
EDNRB	Recurrent	Sedimentation	15	25	Ta-T1	No recurrence	qMSP								20	80			2008
WIF1	Recurrent	Sedimentation	15	25	Ta-T1	No recurrence	qMSP								20	76			2008
TNFRSF25	Recurrent	Sedimentation	15	25	Ta-T1	No recurrence	qMSP								40	56			2008
IGFBP	Recurrent	Sedimentation	15	25	Ta-T1	No recurrence	qMSP								20	84			2008
RASSF1A, ECAD, APC, DAPK, MGMT, BCL2, TERT, EDNRB, WIF1, TNFRSF25. IGFBP	Recurrent	Sedimentation	15	25	Ta-T1	No recurrence	qMSP								86	8	0.448		2008
SALL3, CFTR, ABCC6, HPR1, RASSF1A, MT1A, ALX4, CDH13, RPRM, MINT1, BRCA1	Primary	Sedimentation	132	23/7	Stage 0a–IV	Mixed urologic diseases/healthy	MSP								91.7	87.0		[17]	2007
SALL3	Primary	Sedimentation	132	23/7	Stage 0a–IV	Mixed urologic diseases/healthy	MSP	45.6	72.0	100.0	100.0				58.3	100/100			2007
CFTR	Primary	Sedimentation	132	23/7	Stage 0a–IV	Mixed urologic diseases/healthy	MSP	52.9	52.0	100.0	100.0				55.3	100/100			2007
ABCC6	Primary	Sedimentation	132	23/7	Stage 0a–IV	Mixed urologic	MSP	27.9	250.0	50.0	50.0				36.4	100/100			2007

						diseases/healthy								
HPR1	Primary	Sedimentation	132	23/7	Stage 0a–IV	Mixed urologic diseases/healthy	MSP	32.4	42.0	0.0	50.0	34.8	100/100	2007
BCL2	Primary	Sedimentation	132	23/7	Stage 0a–IV	Mixed urologic diseases/healthy	MSP	22.1	34.0	0.0	50.0	27.3	100/100	2007
ALX4	Primary	Sedimentation	132	23/7	Stage 0a–IV	Mixed urologic diseases/healthy	MSP	22.1	24.0	50.0	0.0	25	100/100	2007
RUNX3	Primary	Sedimentation	132	23/7	Stage 0a–IV	Mixed urologic diseases/healthy	MSP	25.0	44.0	25.0	0.0	32.6	95.7/100	2007
ITGA4	Primary	Sedimentation	132	23/7	Stage 0a–IV	Mixed urologic diseases/healthy	MSP	23.5	42.0	50.0	50.0	31.1	95.7/100	2007
RASSF1A	Primary	Sedimentation	132	23/7	Stage 0a–IV	Mixed urologic diseases/healthy	MSP	27.9	50.0	25.0	100.0	35.6	91.3/100	2007
MYOD1	Primary	Sedimentation	132	23/7	Stage 0a–IV	Mixed urologic diseases/healthy	MSP	17.6	30.0	0.0	50.0	22	100/100	2007
MT1A	Primary	Sedimentation	132	23/7	Stage 0a–IV	Mixed urologic diseases/healthy	MSP	32.4	42.0	25.0	50.0	34.8	91.3/100	2007
DRM	Primary	Sedimentation	132	23/7	Stage 0a–IV	Mixed urologic diseases/healthy	MSP	22.1	18.0	25.0	0.0	18.9	100/100	2007
BMP3B	Primary	Sedimentation	132	23/7	Stage 0a–IV	Mixed urologic diseases/healthy	MSP	13.2	22.0	25.0	0.0	15.9	100/100	2007
CCNA1	Primary	Sedimentation	132	23/7	Stage 0a–IV	Mixed urologic diseases/healthy	MSP	10.3	24.0	25.0	0.0	15.9	100/100	2007
CDH13	Primary	Sedimentation	132	23/7	Stage 0a–IV	Mixed urologic diseases/healthy	MSP	17.6	18.0	25.0	0.0	16.7	100/100	2007
RPRM	Primary	Sedimentation	132	23/7	Stage 0a–IV	Mixed urologic diseases/healthy	MSP	13.2	14.0	50.0	0.0	14.4	100/100	2007
MINT1	Primary	Sedimentation	132	23/7	Stage 0a–IV	Mixed urologic	MSP	8.8	14.0	25.0	50.0	12.9	100/100	2007

							diseases/healthy													
BRCA1	Primary		Sedimentation	132	23/7	Stage 0a–IV	Mixed urologic diseases/healthy	MSP	10.3	16.0	25.0	0.0					12.1	100/100		2007
PTCHD2	Primary		Sedimentation	132	23/7	Stage 0a–IV	Mixed urologic diseases/healthy	MSP	5.9	4.0	25.0	0.0					5.3	100/100		2007
TMS1	Primary		Sedimentation	132	23/7	Stage 0a–IV	Mixed urologic diseases/healthy	MSP	2.9	4.0	0.0	0.0					3	100/100		2007
GSTP1	Primary		Sedimentation	132	23/7	Stage 0a–IV	Mixed urologic diseases/healthy	MSP	2.9	2.0	0.0	0.0					2.3	100/100		2007
P14	Primary	Training	Sedimentation	8	10	HG	Benign	MSP							50			25		2006
P16	Primary	Training	Sedimentation	8	10	HG	Benign	MSP							57			62		2006
RASSF1	Primary	Training	Sedimentation	8	10	HG	Benign	MSP							86			44		2006
APC	Primary	Training	Sedimentation	8	10	HG	Benign	MSP							86			20		2006
GSTP1	Primary	Training	Sedimentation	8	10	HG	Benign	MSP							50			14		2006
E-cad	Primary	Training	Sedimentation	8	10	HG	Benign	MSP							50			11		2006
Cyclin D2	Primary	Training	Sedimentation	8	10	HG	Benign	MSP							37			0		2006
CMI	Primary	Training	Sedimentation	8	10	HG	Benign	MSP							100			50		2006
P14	Primary	Validation	Sedimentation	32	5	Not specified	Benign	MSP									48	40	[88]	2006
P16	Primary	Validation	Sedimentation	32	5	Not specified	Benign	MSP									63	60		2006
RASSF1	Primary	Validation	Sedimentation	32	5	Not specified	Benign	MSP									59	80		2006
APC	Primary	Validation	Sedimentation	32	5	Not specified	Benign	MSP									55	0		2006
GSTP1	Primary	Validation	Sedimentation	32	5	Not specified	Benign	MSP									16	67		2006
E-cad	Primary	Validation	Sedimentation	32	5	Not specified	Benign	MSP									14	17		2006
Cyclin D2	Primary	Validation	Sedimentation	32	5	Not specified	Benign	MSP									35	0		2006

CMI	Primary	Validation	Sedimentation	32	5	Not specified	Benign	MSP									78	80			2006
miR-9-3, miR124-2, miR-124-3, miR-137	Recurrent		Sedimentation	25	107	Ta-T1	No current recurrence	Pyrosequencing									61.5	74	0.71	[29]	2018
miR-129-2/miR-663a			Sedimentation	49	/25	Papillary, invasive, LG, HG	Healthy	qMSP									83.7	88.0		[89]	2017
miR-137		Training	Sedimentation	86	20	≥Ta, grade 1–3	Cancer free, not further specified	qMSP									77.91	77.78	0.782	[90]	2013
miR-124-2		Training	Sedimentation	86	20	≥Ta, grade 1–3	Cancer free, not further specified	qMSP									69.77	88.89	0.769		2013
miR-124-3		Training	Sedimentation	86	20	≥Ta, grade 1–3	Cancer free, not further specified	qMSP									65.12	97.22	0.805		2013
miR-9-3		Training	Sedimentation	86	20	≥Ta, grade 1–3	Cancer free, not further specified	qMSP									69.41	86.11	0.778		2013
OTX1, ONECUT2, TWIST	Primary		Sedimentation	74	80	Ta-T4, grade 1–3	Benign causes of hematuria	SNaPshot and MSP											0.900	[91]	2016
OTX1	Primary		Sedimentation	74	80	Ta-T4, grade 1–3	Benign causes of hematuria	SNaPshot									69.2	62	0.656		2016
ONECUT2	Primary		Sedimentation	74	80	Ta-T4, grade 1–3	Benign causes of hematuria	SNaPshot									77.9	81.8	0.799		2016
TWIST	Primary		Sedimentation	74	80	Ta-T4, grade 1–3	Benign causes of hematuria	MSP									70	89.7	0.799		2016
APC_a, TERT_a, TERT_b, EDNRB	Recurrent	Training	Sedimentation	68	91	Ta-T1, grade 1–3	Non-BC urine	MS-MLPA											0.82	[27]	2012
APC_a, TERT_a, TERT_b, EDNRB	Recurrent	Validation	Sedimentation	49	60	Ta-T1, grade 1–3	No recurrence	MS-MLPA	53	100	100			51	100		63	58	0.69		2012
APC_a, TERT_a, TERT_b, EDNRB	Recurrent	Validation	Sedimentation	65	29	Ta-T4, grade 0-3	No recurrence	MS-MLPA	71	100	83			68	100		72	55			2012
RASSF1A	Primary		Sedimentation	14	10	≥Ta, grade 1–3	Normal, not further specified	MSP						40	55.6		50	100		[92]	2003

MSP = methylation specific PCR, qMSP = quantitative MSP, HG = high grade, LG = low grade, PUNLMP = papillary urothelial neoplasm of low malignant potential, NMIBC = nonmuscle invasive bladder cancer, MIBC = muscle invasive bladder cancer, BC = bladder cancer, MS-MLPA = methylation-specific multiplex ligation-dependent probe amplification, SNUPE = single-nucleotide primer extension, PIN = prostatic intraepithelial neoplasia, DRE = digital rectal examination, GS = Gleason score, MS-HRM = methylation-sensitive high-resolution melting.

Supplementary Table S2. Prostate cancer.

Biomarkers	Urine Collection	Sample Processing	Cancer (n)	Controls (n)	Pathology	Control Population	Method	Overall			Ref.	Year
								Sens. (%)	Spec. (%)	AUC (%)		
PCDH17, TCF21		Sedimentation	50	48	GS 6–9	Healthy	qMSP	26	100	0.650	[39]	2011
PCDH17		Sedimentation	50	48	GS 6–9	Healthy	qMSP	12	100			2011
TCF21		Sedimentation	50	48	GS 6–9	Healthy	qMSP	20	100			2011
GDF15	Morning	Sedimentation	20	20	Not specified	Healthy	qMSP	20	100		[16]	2010
HSPA2	Morning	Sedimentation	20	0	Not specified	Healthy	qMSP	15	100			2010
TMEFF2	Morning	Sedimentation	20	0	Not specified	Healthy	qMSP	0	100			2010
VIM	Morning	Sedimentation	20	0	Not specified	Healthy	qMSP	5	100			2010
APC	Post DRE	Sedimentation	145	123	GS 6–10	Benign	qMSP			0.617	[93]	2018
HOXD3	Post DRE	Sedimentation	145	123	GS 6–10	Benign	qMSP			0.718		2018
TGFbeta2	Post DRE	Sedimentation	145	123	GS 6–10	Benign	qMSP			0.596		2018
GSTP1	Post DRE	Sedimentation	145	123	GS 6–10	Benign	qMSP			0.605		2018
KLK10	Post DRE	Sedimentation	145	123	GS 6–10	Benign	qMSP			0.552		2018
TBX15	Post DRE	Sedimentation	145	123	GS 6–10	Benign	qMSP			0.502		2018
Procure	Post DRE	Sedimentation	145	123	GS 6–10	Benign	qMSP			0.730		2018
GSTP1, RARβ2, APC	Morning	Sedimentation	87	32	T2-T3b	Asymptomatic donors	qMSP	94.3	84.4		[33]	2018
miR-34b/c, miR-193b	Morning	Sedimentation	87	32	T2-T3b	Asymptomatic donors	qMSP	95.4	84.4			2018
GSTP1, RARβ2, APC, miR-34b/c, miR-193b	Morning	Sedimentation	87	32	T2-T3b	Asymptomatic donors	qMSP	100.0	75.0			2018
ADCY4	Post DRE	Sedimentation	38	49	GS 6–10	Negative biopsy results	Nested qMSP	61	78		[36]	2018
ADCY4	First Void	Sedimentation	32	35	GS 6–10	Negative biopsy results	Nested qMSP	56	69			2018
AOX1rc	Post DRE	Sedimentation	38	49	GS 6–10	Negative biopsy results	Nested qMSP	71	69			2018
AOX1rc	First Void	Sedimentation	32	35	GS 6–10	Negative biopsy results	Nested qMSP	44	83			2018
APC2	Post DRE	Sedimentation	38	49	GS 6–10	Negative biopsy results	Nested qMSP	26	90			2018

APC2	First Void	Sedimentation	32	35	GS 6–10	Negative biopsy results	Nested qMSP	13	91		2018
CXCL14	Post DRE	Sedimentation	38	49	GS 6–10	Negative biopsy results	Nested qMSP	21	100		2018
CXCL14	First Void	Sedimentation	32	35	GS 6–10	Negative biopsy results	Nested qMSP	28	97		2018
CXCL14rc	Post DRE	Sedimentation	38	49	GS 6–10	Negative biopsy results	Nested qMSP	16	100		2018
CXCL14rc	First Void	Sedimentation	32	35	GS 6–10	Negative biopsy results	Nested qMSP	13	100		2018
CXCL14 Comb	Post DRE	Sedimentation	38	49	GS 6–10	Negative biopsy results	Nested qMSP	24	100		2018
CXCL14 Comb	First Void	Sedimentation	32	35	GS 6–10	Negative biopsy results	Nested qMSP	34	97		2018
EPHX3	Post DRE	Sedimentation	38	49	GS 6–10	Negative biopsy results	Nested qMSP	66	71		2018
EPHX3	First Void	Sedimentation	32	35	GS 6–10	Negative biopsy results	Nested qMSP	56	69		2018
KIFC2	Post DRE	Sedimentation	38	49	GS 6–10	Negative biopsy results	Nested qMSP	66	78		2018
KIFC2	First Void	Sedimentation	32	35	GS 6–10	Negative biopsy results	Nested qMSP	56	80		2018
KIFC2rc	Post DRE	Sedimentation	38	49	GS 6–10	Negative biopsy results	Nested qMSP	53	86		2018
KIFC2rc	First Void	Sedimentation	32	35	GS 6–10	Negative biopsy results	Nested qMSP	34	91		2018
KIFC2 Comb	Post DRE	Sedimentation	38	49	GS 6–10	Negative biopsy results	Nested qMSP	79	69		2018
KIFC2 Comb	First Void	Sedimentation	32	35	GS 6–10	Negative biopsy results	Nested qMSP	66	77		2018
GFRA2	Post DRE	Sedimentation	38	49	GS 6–10	Negative biopsy results	Nested qMSP	45	84		2018
GFRA2	First Void	Sedimentation	32	35	GS 6–10	Negative biopsy results	Nested qMSP	41	83		2018
GSTP1	Post DRE	Sedimentation	38	49	GS 6–10	Negative biopsy results	Nested qMSP	47	82		2018
GSTP1	First Void	Sedimentation	32	35	GS 6–10	Negative biopsy results	Nested qMSP	47	83		2018
HEMK1	Post DRE	Sedimentation	38	49	GS 6–10	Negative biopsy results	Nested qMSP	39	94		2018
HEMK1	First Void	Sedimentation	32	35	GS 6–10	Negative biopsy results	Nested qMSP	25	91		2018
HOXA7	Post DRE	Sedimentation	38	49	GS 6–10	Negative biopsy results	Nested qMSP	84	80		2018
HOXA7	First Void	Sedimentation	32	35	GS 6–10	Negative biopsy results	Nested qMSP	66	66		2018
HOXB5	Post DRE	Sedimentation	38	49	GS 6–10	Negative biopsy results	Nested qMSP	76	82		2018

HOXB5	First Void	Sedimentation	32	35	GS 6–10	Negative biopsy results	Nested qMSP	72	66		2018
HOXB5rc	Post DRE	Sedimentation	38	49	GS 6–10	Negative biopsy results	Nested qMSP	71	71		2018
HOXB5rc	First Void	Sedimentation	32	35	GS 6–10	Negative biopsy results	Nested qMSP	69	66		2018
HOXB5 Comb	Post DRE	Sedimentation	38	49	GS 6–10	Negative biopsy results	Nested qMSP	84	59		2018
HOXB5 Comb	First Void	Sedimentation	32	35	GS 6–10	Negative biopsy results	Nested qMSP	88	57		2018
HOXD3a	Post DRE	Sedimentation	38	49	GS 6–10	Negative biopsy results	Nested qMSP	50	92		2018
HOXD3a	First Void	Sedimentation	32	35	GS 6–10	Negative biopsy results	Nested qMSP	47	86		2018
HOXD3b	Post DRE	Sedimentation	38	49	GS 6–10	Negative biopsy results	Nested qMSP	76	76		2018
HOXD3b	First Void	Sedimentation	32	35	GS 6–10	Negative biopsy results	Nested qMSP	97	60		2018
HOXD9	Post DRE	Sedimentation	38	49	GS 6–10	Negative biopsy results	Nested qMSP	68	59		2018
HOXD9	First Void	Sedimentation	32	35	GS 6–10	Negative biopsy results	Nested qMSP	63	71		2018
HOXD10	Post DRE	Sedimentation	38	49	GS 6–10	Negative biopsy results	Nested qMSP	61	86		2018
HOXD10	First Void	Sedimentation	32	35	GS 6–10	Negative biopsy results	Nested qMSP	53	77		2018
MOXD1	Post DRE	Sedimentation	38	49	GS 6–10	Negative biopsy results	Nested qMSP	42	84		2018
MOXD1	First Void	Sedimentation	32	35	GS 6–10	Negative biopsy results	Nested qMSP	47	91		2018
NEUROG3	Post DRE	Sedimentation	38	49	GS 6–10	Negative biopsy results	Nested qMSP	37	86		2018
NEUROG3	First Void	Sedimentation	32	35	GS 6–10	Negative biopsy results	Nested qMSP	22	94		2018
NODAL	Post DRE	Sedimentation	38	49	GS 6–10	Negative biopsy results	Nested qMSP	63	82		2018
NODAL	First Void	Sedimentation	32	35	GS 6–10	Negative biopsy results	Nested qMSP	50	80		2018
NODALrc	Post DRE	Sedimentation	38	49	GS 6–10	Negative biopsy results	Nested qMSP	53	84		2018
NODALrc	First Void	Sedimentation	32	35	GS 6–10	Negative biopsy results	Nested qMSP	31	80		2018
NODAL Comb	Post DRE	Sedimentation	38	49	GS 6–10	Negative biopsy results	Nested qMSP	79	71		2018
NODAL Comb	First Void	Sedimentation	32	35	GS 6–10	Negative biopsy results	Nested qMSP	59	69		2018
RASSF5	Post DRE	Sedimentation	38	49	GS 6–10	Negative biopsy results	Nested qMSP	24	94		2018

RASSF5	First Void	Sedimentation	32	35	GS 6–10	Negative biopsy results	Nested qMSP	28	100			2018
RASSF5rc	Post DRE	Sedimentation	38	49	GS 6–10	Negative biopsy results	Nested qMSP	26	88			2018
RASSF5rc	First Void	Sedimentation	32	35	GS 6–10	Negative biopsy results	Nested qMSP	34	86			2018
RASSF5 Comb	Post DRE	Sedimentation	38	49	GS 6–10	Negative biopsy results	Nested qMSP	45	82			2018
RASSF5 Comb	First Void	Sedimentation	32	35	GS 6–10	Negative biopsy results	Nested qMSP	59	86			2018
>= 6 positive of 19 markers	Post DRE	Sedimentation	38	49	GS 6–10	Negative biopsy results	Nested qMSP	89	71			2018
>= 6 positive of 19 markers	First Void	Sedimentation	32	35	GS 6–10	Negative biopsy results	Nested qMSP	94	71			2018
GSTP1	Post DRE	Filtration (8 µm)	38	20	GS 6–10	Negative biopsy results	qMSP	41.9	82.4		[49]	2018
APC	Post DRE	Filtration (8 µm)	38	20	GS 6–10	Negative biopsy results	qMSP	41.9	94.1			2018
RASSF1A	Post DRE	Filtration (8 µm)	38	20	GS 6–10	Negative biopsy results	qMSP	64.5	35.3			2018
PITX2	Post DRE	Filtration (8 µm)	38	20	GS 6–10	Negative biopsy results	qMSP	16.1	100			2018
C1orf114	Post DRE	Filtration (8 µm)	38	20	GS 6–10	Negative biopsy results	qMSP	25.8	100			2018
GSTP1, APC, RASSF1A, PITX2, C1orf114	Post DRE	Filtration (8 µm)	38	20	GS 6–10	Negative biopsy results	qMSP	80.7	17.65			2018
GSTP1	Pre DRE	Filtration (8 µm)	74	25	GS 6–10	Negative biopsy results	qMSP	21.3	76.5			2018
APC	Pre DRE	Filtration (8 µm)	74	25	GS 6–10	Negative biopsy results	qMSP	17.0	94.1			2018
RASSF1A	Pre DRE	Filtration (8 µm)	74	25	GS 6–10	Negative biopsy results	qMSP	46.8	41.2			2018
PITX2	Pre DRE	Filtration (8 µm)	74	25	GS 6–10	Negative biopsy results	qMSP	4.3	100			2018
C1orf114	Pre DRE	Filtration (8 µm)	74	25	GS 6–10	Negative biopsy results	qMSP	6.4	100			2018
GSTP1, APC, RASSF1A, PITX2, C1orf114	Pre DRE	Filtration (8 µm)	74	25	GS 6–10	Negative biopsy results	qMSP	59.6	29.4			2018
miR-34b/c	No DRE	Sedimentation	95	46	≥GS 6	No urological malignancy, healthy	qMSP	89.5	47.8	0.69	[37]	2017
miR-193b	No DRE	Sedimentation	95	46	≥GS 6	No urological malignancy, healthy	qMSP	91.6	95.7	0.96		2017

miR-34b/c+ miR-193b	No DRE	Sedimentation	95	46	≥GS 6	No urological malignancy, healthy	qMSP	90.5	97.8	0.97		2017
cg05163709	Post DRE	Sedimentation	62	73	Not specified	Not specified	Pyrosequncing	94.6	78.3	0.915	[94]	2015
cg27539833 (hypomethylated)	Post DRE	Sedimentation	62	73	Not specified	Not specified	Pyrosequncing	75	70.3	0.729		2015
GSTP1A			5	4	GS 6	Negative biopsy result	qMSP	40	100		[95]	2015
GSTP1B			5	4	GS 6	Negative biopsy result	qMSP	20	100			2015
RASSF1A			5	4	GS 6	Negative biopsy result	qMSP	60	25			2015
RASSF1B			5	4	GS 6	Negative biopsy result	qMSP	60	50			2015
APCA			5	4	GS 6	Negative biopsy result	qMSP	0	100			2015
APCB			5	4	GS 6	Negative biopsy result	qMSP	0	100			2015
DLEC1	Post DRE	Sedimentation	30	30	Not specified	BPH	MSP	36.7	NA		[96]	2015
RARB, GSTP1, RASSF1		Sedimentation	253	32	Not specified	BPH	qMSP	60.1	NA		[97]	2014
RASSF1		Sedimentation	253	32	Not specified	BPH	qMSP	44.7	84.4			2014
RARβ		Sedimentation	253	32	Not specified	BPH	qMSP	29.2	81.3			2014
GSTP1		Sedimentation	253	32	Not specified	BPH	qMSP	11.1	96.9			2014
APC	Post DRE	Sedimentation	10	5	Organ confined	Cancer free (not further specified)	qMSP	50	60		[34]	2014
HOXD3	Post DRE	Sedimentation	10	5	Organ confined	Cancer free (not further specified)	qMSP	100	100			2014
TGFB2	Post DRE	Sedimentation	10	5	Organ confined	Cancer free (not further specified)	qMSP	30	100			2014
TGFB2, HOXD3, APC	Post DRE	Sedimentation	10	5	Organ confined	Cancer free (not further specified)	qMSP	100	60			2014
GSTP1	Post DRE	Sedimentation	14	52	Biopsy positive	Biopsy negative	MSP	42.9	76.9		[98]	2013
RASSF1A	Post DRE	Sedimentation	14	52	Biopsy positive	Biopsy negative	MSP	42.9	71.2			2013
GSTP1	Catheter	Sedimentation	34		GS 6–7		qMSP	3	NA		[99]	2011
RARβ	Catheter	Sedimentation	34		GS 6–7		qMSP	44	NA			2011
RASSF1	Catheter	Sedimentation	34		GS 6–7		qMSP	71	NA			2011

RASSF1, GSTP1, RARβ	Catheter	Sedimentation	34		GS 6–7		qMSP	82	NA			2011
GSTP1, RASSF1A	Post prostatic massage	Sedimentation	34	79	GS 6–10	Negative biopsy	MSP	53.3	45.9	0.788	[100]	2010
GSTP1	Post DRE	Sedimentation	91	50/51	GS 4–9	Young asymptomatic/Biopsy negative	qMSP	81	94/41		[101]	2009
RASSF2	Post DRE	Sedimentation	91	50/51	GS 4–9	Young asymptomatic/Biopsy negative	qMSP	59	63/18			2009
HIST1H4K	Post DRE	Sedimentation	91	50/51	GS 4–9	Young asymptomatic/Biopsy negative	qMSP	92	86/16			2009
TFAP2E	Post DRE	Sedimentation	91	50/51	GS 4–9	Young asymptomatic/Biopsy negative	qMSP	100	18/0			2009
GSTP1	Post DRE	Sedimentation	24	69	<6-10	BPH	qMSP	75	98		[102]	2008
RASSF1a	Post prostatic massage	Sedimentation	95	38	GS 4–10	Negative biopsy	qMSP	77.9	92.1	0.85	[103]	2007
CDH1	Post prostatic massage	Sedimentation	95	38	GS 4–10	Negative biopsy	qMSP	30.5	94.7			2007
APC	Post prostatic massage	Sedimentation	95	38	GS 4–10	Negative biopsy	qMSP	50.5	94.7	0.74		2007
DAPK	Post prostatic massage	Sedimentation	95	38	GS 4–10	Negative biopsy	qMSP	28.4	94.7			2007
MGMT	Post prostatic massage	Sedimentation	95	38	GS 4–10	Negative biopsy	qMSP	14.7	97.4			2007
p16	Post prostatic massage	Sedimentation	95	38	GS 4–10	Negative biopsy	qMSP	11.6	94.7			2007
p14	Post prostatic massage	Sedimentation	95	38	GS 4–10	Negative biopsy	qMSP	6.3	97.4			2007
GSTP1	Post prostatic massage	Sedimentation	95	38	GS 4–10	Negative biopsy	qMSP	83.2	86.8	0.86		2007
RARβ2	Post prostatic massage	Sedimentation	95	38	GS 4–10	Negative biopsy	qMSP	62.1	97.4	0.8		2007
TIMP3	Post prostatic massage	Sedimentation	95	38	GS 4–10	Negative biopsy	qMSP	43.2	100			2007

GSTP1, RASSF1a, RARβ2, APC	Post prostatic massage	Sedimentation	95	38	GS 4–10	Negative biopsy	qMSP	86	NA			2007
APC		Sedimentation	52	91	GS 4–10	No history of genitourinary diseases	qMSP	48	96		[104]	2005
ARF		Sedimentation	52	91	GS 4–10	No history of genitourinary diseases	qMSP	37	100			2005
CDH1		Sedimentation	52	91	GS 4–10	No history of genitourinary diseases	qMSP	77	94			2005
GSTP1		Sedimentation	52	91	GS 4–10	No history of genitourinary diseases	qMSP	48	100			2005
MGMT		Sedimentation	52	91	GS 4–10	No history of genitourinary diseases	qMSP	19	100			2005
p16		Sedimentation	52	91	GS 4–10	No history of genitourinary diseases	qMSP	37	100			2005
RAR-2		Sedimentation	52	91	GS 4–10	No history of genitourinary diseases	qMSP	35	91			2005
RASSF1A		Sedimentation	52	91	GS 4–10	No history of genitourinary diseases	qMSP	73	89			2005
TIMP3		Sedimentation	52	91	GS 4–10	No history of genitourinary diseases	qMSP	37	91			2005
p16, ARF, MGMT, GSTP1		Sedimentation	52	91	GS 4–10	No history of genitourinary diseases	qMSP	87	100			2005
GSTP1	Post prostatic massage		40	45	Intracapsular cancer and locally advanced or systemic cancer	BPH	MSP	73	98		[105]	2001
APC	Post DRE	Sedimentation	97	83	GS 6–9	BPH, PIN, Atypia	qMSP			0.585	[106]	2009
GSTP1	Post DRE	Sedimentation	97	83	GS 6–9	BPH, PIN, Atypia	qMSP			0.664		2009
RARβ2	Post DRE	Sedimentation	97	83	GS 6–9	BPH, PIN, Atypia	qMSP			0.705		2009
RARβ2, GSTP1, APC	Post DRE	Sedimentation	97	83	GS 6–9	BPH, PIN, Atypia	qMSP	60	81	0.72		2009
GSTP1	Post DRE	Sedimentation	54	67	GS 4–10	Biopsy negative	qMSP	33	95	0.65	[107]	2008

RARβ	Post DRE	Sedimentation	54	67	GS 4–10	Biopsy negative	qMSP	40	84	0.59		2008
APC	Post DRE	Sedimentation	54	67	GS 4–10	Biopsy negative	qMSP	36	91	0.59		2008
GSTP1, APC	Post DRE	Sedimentation	54	67	GS 4–10	Biopsy negative	qMSP	51	89	0.68		2008
GSTP1, APC, RARβ	Post DRE	Sedimentation	54	67	GS 4–10	Biopsy negative	qMSP	55	80	0.69		2008
GSTP1	Post DRE	Sedimentation	57	56	GS 4–10	Biopsy negative	qMSP	36	91	0.64		2008
RARβ	Post DRE	Sedimentation	57	56	GS 4–10	Biopsy negative	qMSP	29	91	0.64		2008
APC	Post DRE	Sedimentation	57	56	GS 4–10	Biopsy negative	qMSP	51	83	0.62		2008
GSTP1, APC	Post DRE	Sedimentation	57	56	GS 4–10	Biopsy negative	qMSP	53	80	0.67		2008
GSTP1, APC, RARβ	Post DRE	Sedimentation	57	56	GS 4–10	Biopsy negative	qMSP	53	76	0.65		2008
GSTP1	Post DRE/biopsy	Sedimentation	12	5	GS 6–7	Biopsy Negative	MSP	25/17	80/80		[35]	2006
APC	Post DRE/biopsy	Sedimentation	12	5	GS 6–7	Biopsy Negative	MSP	8/17	80/80			2006
EDNRB	Post DRE/biopsy	Sedimentation	12	5	GS 6–7	Biopsy Negative	MSP	63/88	25/40			2006
EDNRB, APC, GSTP1	Post DRE/biopsy	Sedimentation	12	5	GS 6–7	Biopsy Negative	MSP	100	40			2006
GSTP1	Post biopsy	Sedimentation	18	18	Not specified	Negative Biopsy	MSP	58	39		[108]	2003
GSTP1		Sedimentation	69	31	Not specified	BPH	qMSP	18.8	96.8		[109]	2002
GSTP1		Sedimentation	69	31	Not specified	BPH	MSP	30.4	96.8			2002
GSTP1	Post Prostatic massage		11	10	Not specified	BPH	MSP	36	100		[110]	2001
GSTP1	Post Prostatic massage		29	40	Not specified	BPH	MSP	76	97			2001
GSTP1	Post DRE		65	45	Clinically localized cancer	BPH	MSP	97.8	88.9	0.936	[111]	2013

MSP = methylation specific PCR, qMSP = quantitative MSP, DRE = digital rectal examination, GS = Gleason score, PIN =.prostatic intraepithelial neoplasia, BPH = benign prostate hyperplasia
